# Magnetically Recyclable Wool Keratin Modified Magnetite Powders for Efficient Removal of Cu^2+^ Ions from Aqueous Solutions

**DOI:** 10.3390/nano11051068

**Published:** 2021-04-21

**Authors:** Xinyue Zhang, Yani Guo, Wenjun Li, Jinyuan Zhang, Hailiang Wu, Ningtao Mao, Hui Zhang

**Affiliations:** 1 School of Environmental and Chemical Engineering, Xi′an Polytechnic University, Xi′an, Shaanxi 710048, China; 41504040228@stu.xpu.edu.cn (X.Z.); 19940706@xpu.edu.cn (Y.G.); 2 Research Centre for Functional Textile Materials, School of Textile Science and Engineering, Xi’an Polytechnic University, Xi’an, Shaanxi 710048, China; 200111006@stu.xpu.edu.cn (W.L.); 190121054@stu.xpu.edu.cn (J.Z.); 3 Key Laboratory of Functional Textile Material and Product, Xi’an Polytechnic University, Ministry of Education, Xi’an, Shaanxi 710048, China; whl@xpu.edu.cn; 4 Performance Textiles and Clothing Research Group, School of Design, University of Leeds, Leeds LS2 9JT, UK; n.mao@leeds.ac.uk

**Keywords:** magnetite (Fe_3_O_4_), wool keratin, Cu^2+^ ions, adsorption

## Abstract

The treatment of wastewater containing heavy metals and the utilization of wool waste are very important for the sustainable development of textile mills. In this study, the wool keratin modified magnetite (Fe_3_O_4_) powders were fabricated by using wool waste via a co-precipitation technique for removal of Cu^2+^ ions from aqueous solutions. The morphology, chemical compositions, crystal structure, microstructure, magnetism properties, organic content, and specific surface area of as-fabricated powders were systematically characterized by various techniques including field emission scanning electron microscopy (FESEM), energy dispersive spectroscopy (EDS), X-ray diffraction (XRD), transmission electron microscopy (TEM), X-ray photoelectron spectroscopy (XPS), vibrating sample magnetometer (VSM), thermogravimetric (TG) analysis, and Brunauer–Emmett–Teller (BET) surface area analyzer. The effects of experimental parameters such as the volume of wool keratin hydrolysate, the dosage of powder, the initial Cu^2+^ ion concentration, and the pH value of solution on the adsorption capacity of Cu^2+^ ions by the powders were examined. The experimental results indicated that the Cu^2+^ ion adsorption performance of the wool keratin modified Fe_3_O_4_ powders exhibited much better than that of the chitosan modified ones with a maximum Cu^2+^ adsorption capacity of 27.4 mg/g under favorable conditions (0.05 g powders; 50 mL of 40 mg/L CuSO_4_; pH 5; temperature 293 K). The high adsorption capacity towards Cu^2+^ ions on the wool keratin modified Fe_3_O_4_ powders was primarily because of the strong surface complexation of –COOH and –NH_2_ functional groups of wool keratins with Cu^2+^ ions. The Cu^2+^ ion adsorption process on the wool keratin modified Fe_3_O_4_ powders followed the Temkin adsorption isotherm model and the intraparticle diffusion and pseudo-second-order adsorption kinetic models. After Cu^2+^ ion removal, the wool keratin modified Fe_3_O_4_ powders were easily separated using a magnet from aqueous solution and efficiently regenerated using 0.5 M ethylene diamine tetraacetic acid (EDTA)-H_2_SO_4_ eluting. The wool keratin modified Fe_3_O_4_ powders possessed good regenerative performance after five cycles. This study provided a feasible way to utilize waste wool textiles for preparing magnetic biomass-based adsorbents for the removal of heavy metal ions from aqueous solutions.

## 1. Introduction

It is known that the long-term accumulation of heavy metal ions in biological system can definitely cause serious damage to human, animal, and environment as they are highly toxic and non-biodegradable [1]. In comparison with the conventional methods, such as chemical precipitation, ion exchange, chemical oxidation/reduction, and membrane filtration, the adsorption technique has gained much interest for removing heavy metal ions from aqueous solutions via physisorption or chemisorption mechanisms because of its simplicity and high sorption efficiency [2]. In recent years, the renewable polymer absorbents from biogenic materials have been recognized as the ideal alternatives due to their low cost and abundance [3]. Among the various biosorbents, keratin materials like wool, feathers, and hair can be effectively used for the treatment of heavy metal pollution mainly because they have the polarizable and ionizable groups [4]. In particular, wool keratins possess a large number of functional groups, including sulfhydryl (–SH), amide (–CONH_2_), carboxyl (–COOH), and hydroxyl (–OH) [5], and these groups can interact strongly with heavy metal ions by valence forces, resulting in higher adsorption capacity than other keratinous materials [6]. It has been demonstrated that the adsorption sites on wool keratins are primarily focused on the groups of amino and carboxyl and the cleavage of disulfide bonds [7]. In acidic medium, the surface of wool keratins is positive charged owing to the protonation of amino groups and the dissociation of carboxyl groups. Thus, carboxyl groups can react with metal ions to form metal/wool complex [7].

Wools are a kind of bio-polymeric materials having acidic and basic ampholite sorption centers. The major component of wools is 98% protein by weight and keratins make up 85% of the total protein content. The rest is composed of hydrocarbon and other compounds [8]. The wool keratins are consisted of about 18 kinds of amino acids and have different contents of hydrophobic and hydrophilic groups [9]. By virtue of cost-effective, biodegradable, and no secondary pollution, wool fibers can be applied for the extraction of toxic heavy metal ions from aqueous solutions through in situ adsorption process [10]. The adsorption capability of wools towards heavy metal ions can be improved to certain degrees after being irradiated with accelerated electron beam [11], or blended with polyamide 6 [12], polyethylene [13], and polyethylene terephthalate [14]. In addition, the concentrations of chemical agents of Na_2_S, NaBH_4_, NaOH and NaHSO_3_ have great effects on the adsorption capacities towards metal ions on account of the formation of carboxyl, amino, and thiol groups in wools [15]. It has been confirmed that the wool-based particles [16] or nanofibers [17] show higher sorption capacities towards heavy metal ions than wool fibers. This is attributed to the increased specific surface area and more exposed active sites. However, the separation and recycling of particulate wool adsorbents from solutions restrict its processing and practical applications.

The typical nanomaterial sorbents with magnetic features have been widely studied for the removal of heavy metal species because they have high surface area and are easy to separate from aqueous solutions by applying external magnetic fields [18]. Recently, many magnetite (Fe_3_O_4_) based adsorbents have been developed by incorporation of chitosan [19], chitosan and calcium alginate [20], chitosan and polyether sulfone [21], polyacrylamidoxime [22], asparagine [23], polyethylenimine [24], ethylenediamine coupled with graphene oxide and chitosan-g-poly(acrylic acid-co-2-acrylamido-2-methylpropane sulfonic acid) copolymer [25], TiO_2_ and graphene [26], graphene oxide [27], amino acid and graphene oxide [28], carbon [29], polydopamine or polyamidoamine [30], NH_2_-MIL-125 (Ti) [31], organodisulfide [32], curcumin [33], alginate [34], zeolite and cellulose nanofibers [35], and selective adsorbents ethylene diamine tetraacetic acid [36] and N-(trimethoxysilylpropyl) ethylenediamine triacetic acid [37]. For example, the catechol groups of dopamine can integrate with metal ions and increase the hydrophilic ability of as-prepared adsorbents [38]. The binding between Fe_3_O_4_ and adsorbents can be strengthened using the cross-linker carbodiimide [39].

In addition, the surface functionalization of Fe_3_O_4_ not only solve the aggregation issue, but also introduce various functional groups for better removing of specific heavy metal ions [40]. The adsorption properties of functionalized Fe_3_O_4_ are distinctly affected by the kinds of organic acids used [41]. To date, many functionalized Fe_3_O_4_ particles have been prepared using 3-aminopropyltriethoxysilane and succinic anhydride [42], poly(amic acid) triethylamine salts [43], poly(acrylic acid) [44], L-glutamic acid [45], L-cysteine [46], ammonium hydroxide (3-mercaptopropyl)-trimethoxysilane [47], oleic acid combined with methyl methacrylate, allyl thiourea and ethylenediamine [48], citric acid [49], amino acid [50], amino-functionalized poly(dimethylsiloxane) [51], and amino-guanidinopentanoic acid functionalized benzene dicarboxylic [52].

It has been reported that a large amount of available waste wools is produced from sheep breeding and by-products from textile industry, accounting for more than 5 million tons per year [17]. The adsorption capacity towards heavy metal ions can be greatly improved when fibrous wools are transformed into fine powder form. However, wool powders making is a costly and time-consuming processing. In the present work, in order to make full use of wool waste to treat wastewater containing heavy metal ions, waste wool fibers were employed as the biosorbent to fabricate the magnetically recyclable Fe_3_O_4_ powders modified with wool keratin based on a modified co-precipitation method. The adsorption parameters, such as the powder dosage, the initial two valence copper (Cu^2+^) ion concentration, the pH of solution, and the contact time, for the removal of Cu^2+^ ions by the powders were optimized in batch experiments. The adsorption performance of wool keratin modified Fe_3_O_4_ powders was compared with that of chitosan modified ones. Furthermore, the adsorption properties of the wool keratin modified Fe_3_O_4_ powder towards Cu^2+^ ions were evaluated by applying the thermodynamic and kinetic models. The adsorption mechanism was also proposed. This study might provide a facile and effective way to reuse waste wool products.

## 2. Experimental Section

### 2.1. Materials and Reagents

The chemical reagents used in this work were analytical grade and included sodium carbonate anhydrous (Na_2_CO_3_), iron (III) chloride hexahydrate (FeCl_3_·6H_2_O), iron (II) chloride tetrahydrate (FeCl_2_·4H_2_O), sodium hydroxide (NaOH), acetic acid (CH_3_COOH), citric acid (C_6_H_8_O_7_), sodium hypophosphite (NaH_2_PO_2_), copper sulfate pentahydrate (CuSO_4_·5H_2_O), ammonium chloride (NH_4_Cl), 40% wt.% acetaldehyde (CH_3_CHO), ammonia solution (NH_3_·H_2_O, 28% in water), ammonium citrate ((NH_4_)_3_C_6_H_5_O_7_), ethylene diamine tetraacetic acid (EDTA, C_10_H_16_N_2_O_8_), sulfuric acid (H_2_SO_4_), and absolute ethanol (C_2_H_6_O) from Shanghai Macklin Biochemical Co., Ltd. (Shanghai, China). The cuprizone (bis(cyclohexanone)oxalyldihydrazone, BCO, C_14_H_22_N_4_O_2_) was purchased from Tianjin Kemiou Chemical Reagent Co., Ltd. (Tianjin, China). The silane coupling agent KH550 ((3-aminopropyl)triethoxysilane, C_9_H_23_NO_3_Si) and chitosan with deacetylation degree >90% and weight average molecular weight 30,000 were purchased from Shanghai Macklin Biochemical Co., Ltd. (Shanghai, China). The wool waste fibers with an average diameter of 28 ± 2 µm were collected from Yulin Qinyang Clothing Co., Ltd. (Yulin, China). The deionized water was applied for preparing the solution throughout the experiments.

### 2.2. Fabrication of Fe_3_O_4_ Powders Modified with Wool Keratin

#### 2.2.1. Preparation of Wool Keratin Hydrolysate from Wool Fibers

The as-obtained wool fibers were first pretreated in a weak alkaline solution to remove the grease and other impurities from wool fibers. Typically, according to the ratio of material to liquor 1:80, 3.0 g of wool fibers was dipped into 240 mL of 2.0 g/L sodium carbonate solution at 60 °C for 30 min without stirring, and then washed with deionized water at 40 °C for 10 min thrice. The wool fibers were further immersed in absolute ethanol solution at room temperature for 10 min, and subsequently washed with deionized water until the pH of the solution was neutral, and lastly dried in an oven at 50 °C overnight. 2.0 g of degreased wool fibers was cut into snippets of length less than 5 mm and then immersed in 100 mL of 40 g/L NaOH solution at 80 °C for 6 h under continuous stirring until the wool fibers were dissolved completely. During the dissolving process, the mixture solution was ultrasonically treated at 28 kHz and 50 W for 15 min every 1 h to obtain the wool keratin hydrolysate.

#### 2.2.2. Fabrication of Wool Keratin Modified Fe_3_O_4_ Powders

**Scheme 1:**
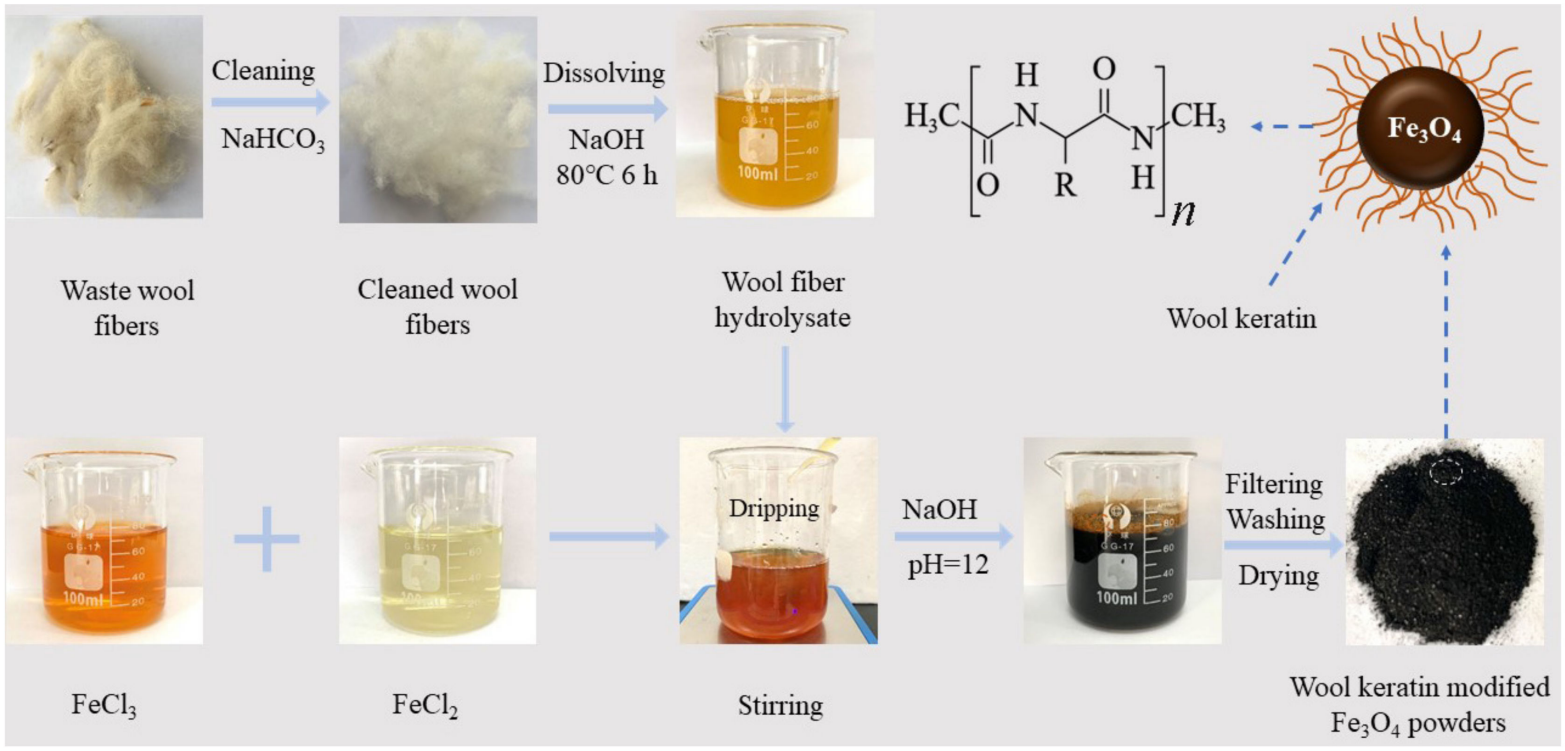
The synthesis route for the fabrication of wool keratin modified Fe_3_O_4_ powders.

According to the co-precipitation method [53], the wool keratin modified Fe_3_O_4_ powders were fabricated by using Fe^3+^ and Fe^2+^ salts in alkaline aqueous solution by adding the wool keratin hydrolysate for in-suit synthesis of Fe_3_O_4_. The detailed route for synthesis of wool keratin modified Fe_3_O_4_ powders is described in Scheme 1.

The disulfide bonds in wools were broken to form mercapto (–SH) groups under basic conditions. The alkaline buffers could cause destruction to the cystine, serine, and threonine of wools to a small extent, and thus their *α*-spiral polypeptide chains were relatively stabilized for a certain time [54] and grafted onto the as-fabricated Fe_3_O_4_. To begin with, a certain amount of FeCl_2_·4H_2_O (1.6 g) and FeCl_3_·6H_2_O (3.7 g) was added into 100 mL deionized water at 25 °C under continuous stirring to attain a mixture solution. Eight identical mixture solutions were accordingly prepared. A series of wool keratin hydrolysate (0, 5, 10, 20, 40, 60, 80, and 100 mL) were separately added dropwise into the eight mixture solutions. Next, 40 mg/L of NaOH solution was subsequently added dropwise in each mixture solution under vigorous stirring to adjust the pH value of solution at 12. The eight mixture solutions were heated to 50 °C in a water bath and maintained at the constant temperature for 2 h until the crystallization of black Fe_3_O_4_ particles was realized. Lastly, the magnetic powders in each solution were separated from the solution by a magnet, and successive washed with absolute ethanol and deionized water thrice, and dried in a vacuum oven at 60 °C for overnight. The optimized wool keratin modified Fe_3_O_4_ powders were chosen for the characterization and the measurements of Cu^2+^ ions adsorption including adsorption isotherms, adsorption kinetics, and thermodynamic studies. For comparison, the chitosan modified Fe_3_O_4_ powders were also fabricated based on the same precipitation method as previous reported [55] and the detailed preparation steps were described in the Supplementary Materials S1.

### 2.3. Characterization Techniques

The morphologies of as-fabricated powders were observed on a Zeiss Evo18 Compact field emission scanning electron microscope (FESEM, Carl Zeiss AG, Oberkochen, Germany), and the elements were analyzed using an Oxford INCA energy dispersive spectrometer (EDS, Oxford Inca Energy 400, Oxford, UK) coupled with the FESEM. The crystal structures of as-fabricated powders were investigated on an XRD-7000S X-ray diffraction (XRD) instrument (Shimadzu Corp., Kyoto, Japan) using Cu K*α*_1_ radiation (*λ* = 0.154056 nm) at 40 kV and 40 mA in the 2*θ* range of 10–80° at a scanning rate of 10°/min. The microstructures of as-fabricated powders were identified on a JEM-3010 transmission electron microscope (TEM, JEOL Ltd., Kyoto, Japan) at 300 kV. The surface chemical compositions of as-fabricated powders were performed on a K-Alpha X-ray photoelectron spectrometer (XPS, Kratos Analytical Ltd., Manchester, UK) with the Al Kα line at 1486.6 eV. The magnetic properties of as-fabricated powders were conducted on a VersaLab multifunction vibrating sample magnetometer (VSM, Quantum Design, San Diego, CA, USA) at a frequency of 40 Hz and an amplitude of 2 mm. The amounts of organic compounds coated on magnetic powders were determined on a TGA Q500 thermogravimetric (TG) analyzer (TA Instrument Co., New Castle, DE, USA) under nitrogen environment at a heating rate of 10 °C/min in 40–600 °C ranges. The adsorption–desorption isotherms and pore volume versus pore size distribution curves of as-fabricated powders were carried out on a surface area analyzer (Micromeritics Gemini VII 2390, Micromeritics Instrument Co., Norcross, GA, USA).

### 2.4. Measurements of Cu^2+^ Ion Adsorption Capacity

The adsorption properties of as-fabricated powders towards heavy metal ions were measured by using Cu^2+^ as the model according to the Chinese standard GB/T6730.35–2016 [56]. In a typical experiment, a certain amount of as-fabricated powders (0.05 g) was added into 50 mL of 10 mg/L CuSO_4_ aqueous solution at specific temperature and then sonicated at 28 kHz and 50 W for 30 min. After 24 h of adsorption–desorption equilibrium, 5 mL supernatant containing of Cu^2+^ ions was collected by centrifugation at a speed of 11,000 rpm. 7 mL of 20% ammonium citrate, 5 mL of buffer solution containing 40 g/L of ammonium chloride and 40 mL/L of ammonia solution, 1 mL of 40% acetaldehyde, and 11 mL of 2 g/L BCO solution were successively added into the as-collected supernatant. Thus, a green color mixture solution was produced because of the color reaction between Cu^2+^ and cuprizone [56]. The volume of the mixture solution was controlled at 50 mL by adding deionized water. By reference to the blank cuprizone solution, the absorbance *A* of the color solution was tested on an ultraviolet–visible spectrophotometer at the maximum absorption wavelength of 545 nm. The concentration *C* (mg/L) of Cu^2+^ ions was calculated on the basis of the standard regression equation (*C* = 2.41 × *A* − 0.02, coefficient of determination *R*^2^ = 0.99). The removal efficiency (*R*_e_, %) and equilibrium adsorption capacity (*q*_e_, mg/g) towards Cu^2+^ ions by the as-fabricated powders were respectively calculated according to the Equation (1) and (2) as below:

(1) $$R_{e}=\frac{C_{0}-C_{e}}{C_{0}}\times 100\%$$

(2) $$q_{e}=\frac{C_{0}-C_{e}}{M}\times V$$

where *C*_0_ and *C*_e_ (mg/L) were the initial and equilibrium concentrations of Cu^2+^ ions respectively; *M* (g) was the adsorbent mass of as-fabricated powders; *V* (L) was the volume of Cu^2+^ ion solution. The effects of the volume of wool keratin hydrolysate, the dosage of powders, the initial Cu^2+^ ion concentration, and the pH value of solution on the adsorption behaviors of wool keratin modified Fe_3_O_4_ powders were systematically examined. The volumes of wool keratin hydrolysate were from 0 to 100 mL. The dosages of powders varied from 0.01 to 0.1 g. The initial Cu^2+^ ion concentrations were in the range of 5–50 mg/L. The pH values of the solutions were controlled from 3 to 9 by adding 0.1 M HCl or NaOH. Moreover, the adsorption behaviors including adsorption isotherms, adsorption kinetics, and adsorption thermodynamics onto the wool keratin modified Fe_3_O_4_ powders were studied at 293, 303, 313 and 323 K, respectively.

Furthermore, the Cu^2+^ desorption experiments were conducted using 0.5 M EDTA-H_2_SO_4_ (volume ratio of 1:1) mixed solution. The Cu^2+^ adsorbed powders were dipped in 30 mL EDTA-H_2_SO_4_ mixed solution for 30 min under continuous stirring, and then washed repeatedly with deionized water at room temperature. The powders after desorption were reused for the next adsorption experiment and the procedure was repeated for five times. All adsorption experiments were conducted at least three times to ensure the accuracy greater than 95% and the average value was given.

## 3. Results and Discussion

### 3.1. Surface Morphology Observation and Element Analysis

**Figure 1:**
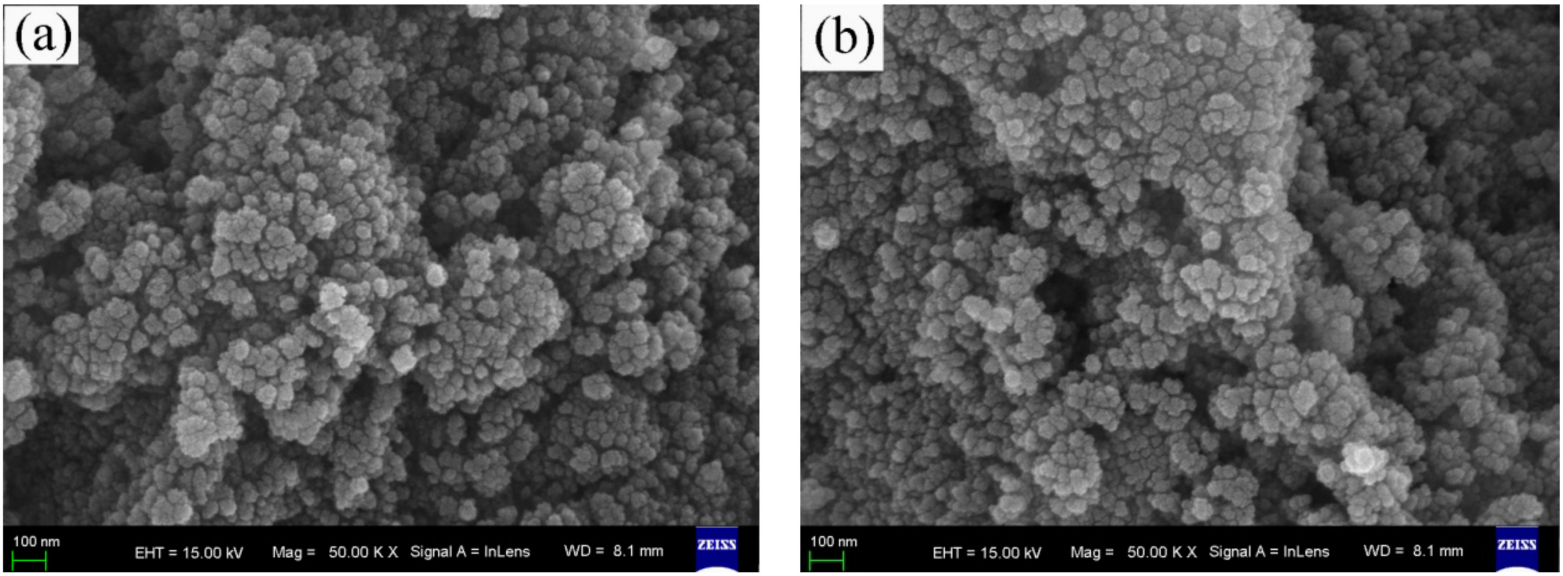

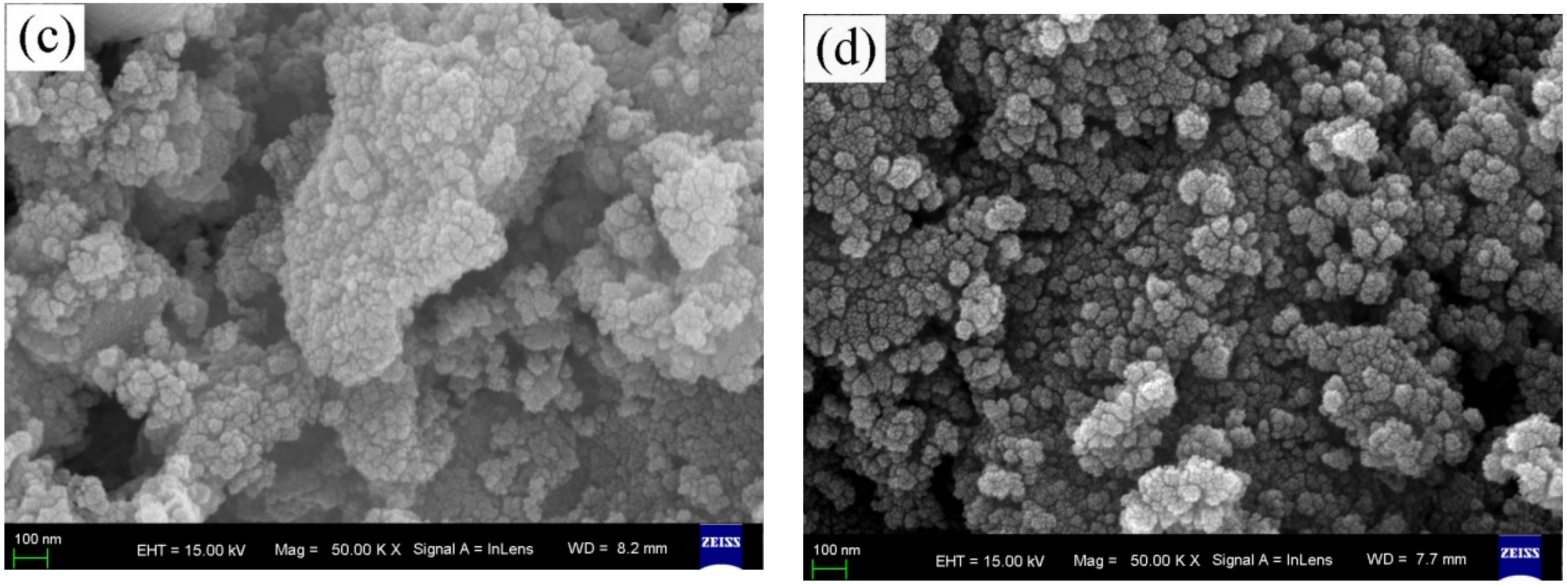
Field emission scanning electron microscopy (FESEM) images of (**a**) pure, (**b**) wool keratin modified, (**c**) KH550 modified, and (**d**) chitosan modified Fe_3_O_4_ powders.

The FESEM images of the surface morphologies of pure, wool keratin modified, KH550 modified, and chitosan modified Fe_3_O_4_ powders were exhibited in Figure 1. It was seen that there was no distinct difference among the four as-fabricated powders. All of them exhibited the spherical appearances with diameter ranges of less than 100 nm to form the particulate aggregates, which were in accordance with the previous studies [57].

The EDS patterns of the pure, wool keratin modified, KH550 modified, and chitosan modified Fe_3_O_4_ powders were illustrated in Supplementary Materials Figure S1. The element analysis results in the inserts in Figure S1 indicated that the four as-fabricated powders contained the elements of C, O, Na and Fe. Obviously, the Fe element was resulted from Fe_3_O_4_ and the Na element was induced by the usage of NaOH. The C element was probably derived from the organic pollutants for the pure Fe_3_O_4_, the amino acids for the wool keratin modified Fe_3_O_4_, the silane coupling agent for the KH550 modified Fe_3_O_4_, and the carbon component of chitosan for the chitosan modified Fe_3_O_4_ powders. The O element was mainly attributed to Fe_3_O_4_. In comparison with the pure Fe_3_O_4_, the elements of C and O in the wool keratin modified Fe_3_O_4_ increased to some degree, implying the introduction of wool keratins. Moreover, the Si element was ascribed to the silane coupling agent KH550 in the KH550 modified Fe_3_O_4_. After treatment with chitosan, the O element decreased while the Fe element increased.

### 3.2. Crystal Structure Analysis

**Figure 2:**
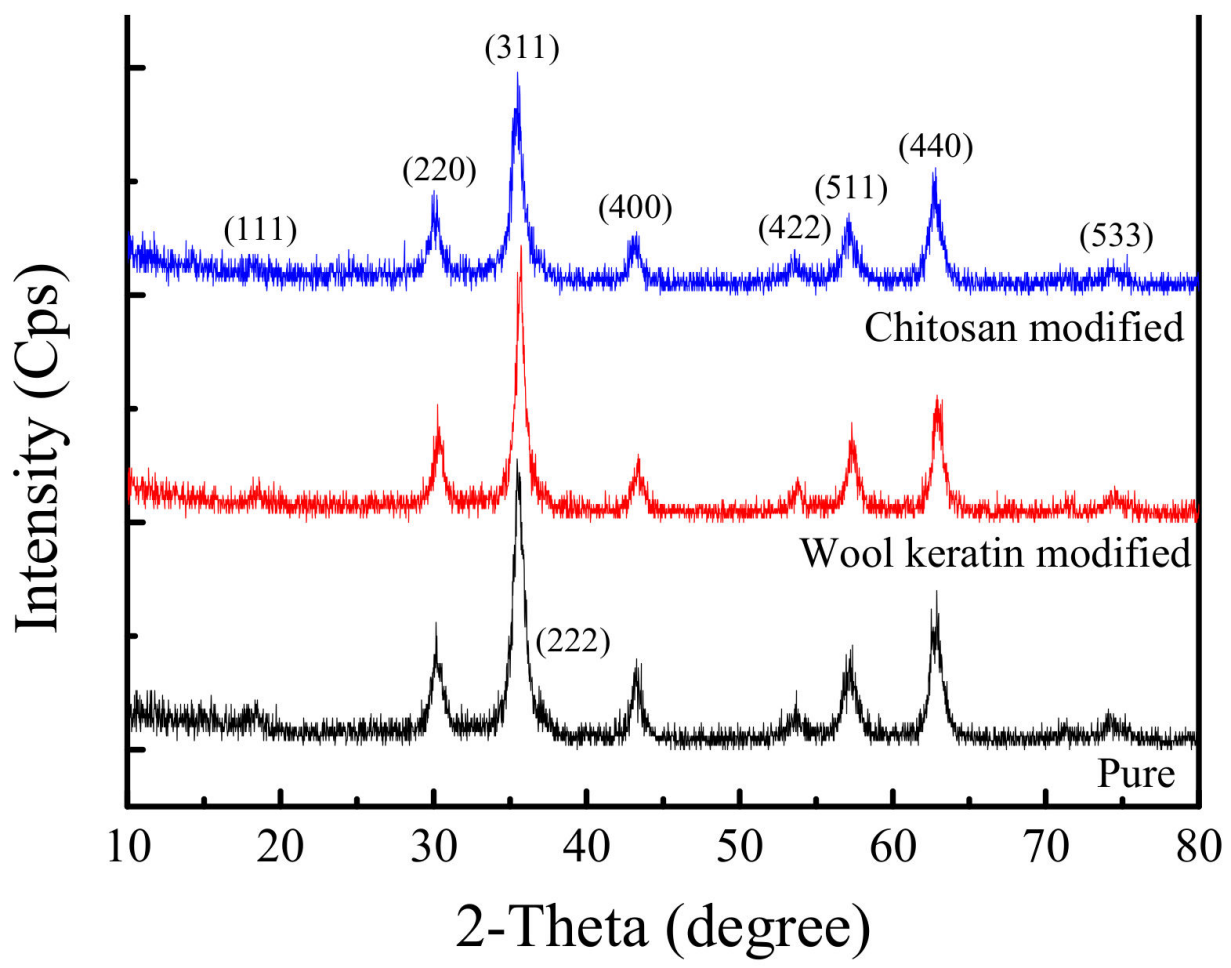
X-ray diffraction (XRD) patterns of the pure, wool keratin modified, and chitosan modified Fe_3_O_4_ powders.

The XRD patterns of the pure, wool keratin modified, and chitosan modified Fe_3_O_4_ powders were represented in Figure 2. It was clear that the three as-fabricated Fe_3_O_4_ powders had the similar XRD pattern. The characteristic diffraction peaks were detected 2*θ* = 18.3°, 30.1°, 35.6°, 43.3°, 53.9°, 57.1°, 62.9° and 74.3°, corresponding to the (111), (220), (311), (400), (422), (511), (440) and (533) planes of inverse cubic spinel structure of magnetite Fe_3_O_4_ (JCPDS Card no.19-0629) [58], respectively. Based on the Scherrer’s Equation (3) [53] as below:

(3) $$D=\frac{K\lambda }{\beta \cos\theta }$$

where *D* was the crystal size, *K* was the Scherrer coefficient (0.89), *λ* was the X-ray wavelength (*λ* = 0.154056 nm), *θ* was the Bragg angle, *β* was the full width at half-maximum (FWHM) in radians, the crystalline sizes of as-fabricated Fe_3_O_4_ powders were respectively calculated to be 11.5, 12.8 and 12.6 nm for the pure, wool keratin modified and chitosan modified Fe_3_O_4_. Thus, the incorporation of wool hydrolysate or chitosan did not affect the crystal structure of Fe_3_O_4_, but the crystalline size increased slightly.

### 3.3. Microstructure Analysis

The TEM and selected area electron diffraction (SAED) images of the pure Fe_3_O_4_ powders were shown in Supplementary Materials Figure S2. It was noticed that the pure Fe_3_O_4_ powders possessed the spherical appearance with an average particle size of around 25 nm. The grain edges of Fe_3_O_4_ particles were clear without any attachment, and the crystallization was insufficient. A series of weak diffraction rings could be found in the SAED pattern, which were indexed to the (111) and (311) crystal planes of magnetite Fe_3_O_4_ [59]. The element analysis results suggested that four elements were detected in pure Fe_3_O_4_ powders. In addition to the elements of O and Fe derived from Fe_3_O_4_, the relatively high amount of C element was probably caused by carbon membrane of copper mesh, and a trace amount of Si element might be polluted by glass beak used.

**Figure 3:**
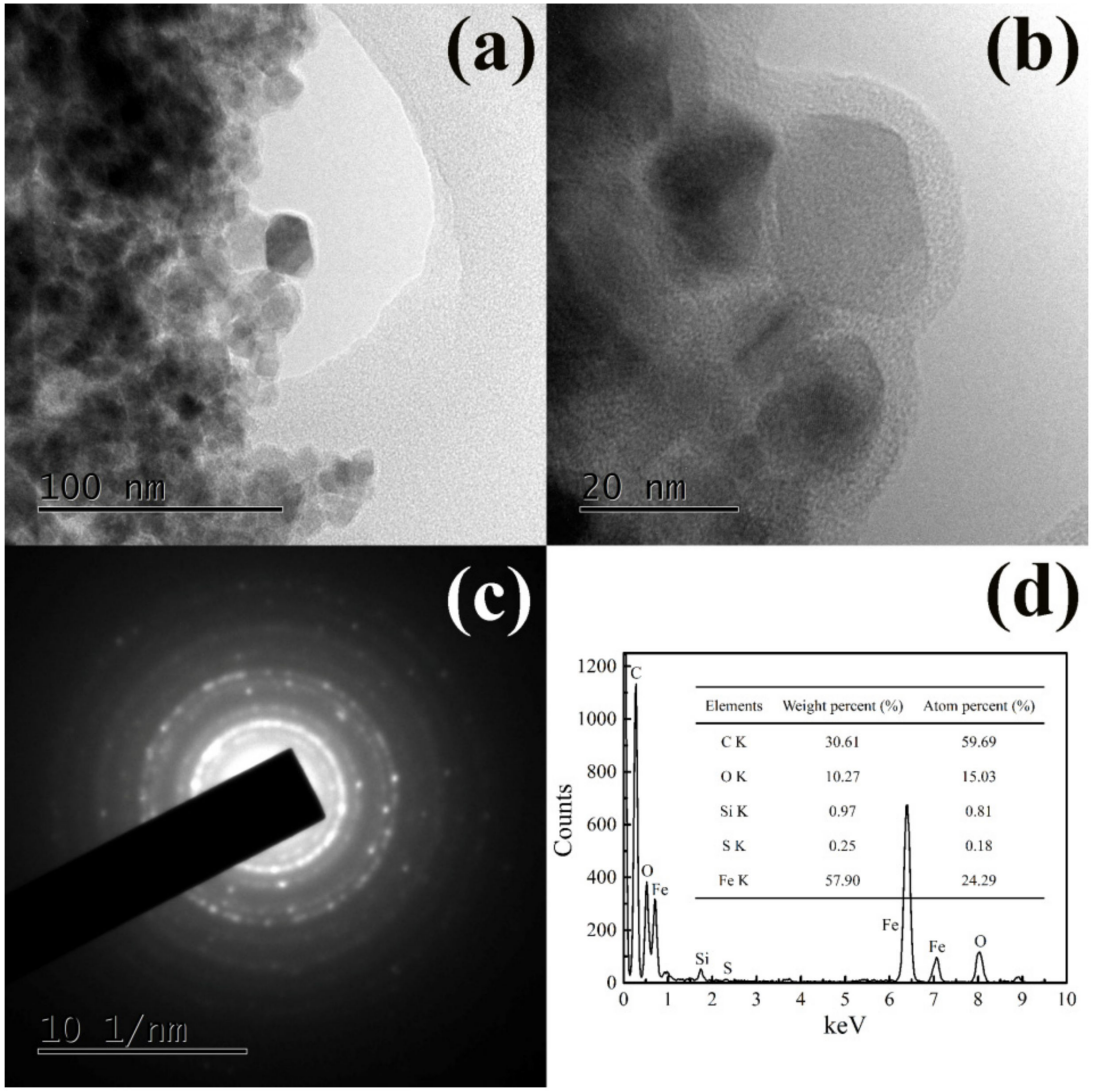
The (**a**) and (**b**) transmission electron microscopy (TEM), and (**c**) selected area electron diffraction (SAED) images, and (**d**) energy dispersive X-ray (EDX) ( spectrum along with element analysis data for the wool keratin modified Fe_3_O_4_ powders.

The TEM and SAED images of the wool keratin modified Fe_3_O_4_ powders were presented in Figure 3. The average particle size of the wool keratin modified Fe_3_O_4_ powders was the same as that of pure Fe_3_O_4_. Meanwhile, a few long rod-like substances were found, which might be the undissolved cortical cells modified with Fe_3_O_4_. Importantly, the wool keratin modified Fe_3_O_4_ powders were completely coated by a layer of organic matter. The strong diffraction rings in SAED image confirmed the well crystallization of wool keratin modified Fe_3_O_4_ powders. In addition, besides the elements of C, O, Si and Fe, the S element was detected, implying the presence of wool keratins.

The TEM and SAED images of the chitosan modified Fe_3_O_4_ powders were revealed in Supplementary Materials Figure S3. There was no distinct change in appearance for both the wool keratin modified and chitosan modified Fe_3_O_4_ powders. Similarly, some organic substances were deposited on the surface of the chitosan modified Fe_3_O_4_ powders. The SAED image showed the high crystallinity of the chitosan modified Fe_3_O_4_. Except the elements of C, O, Si, and Fe, no other elements were detected. The increase of the content of Si element was attributed to the modification of KH550. However, the N element of chitosan was not detected probably because of the small amount of chitosan onto Fe_3_O_4_ powders and the high deacetylation degree of chitosan.

### 3.4. Surface Chemical Composition Analysis

**Figure 4:**
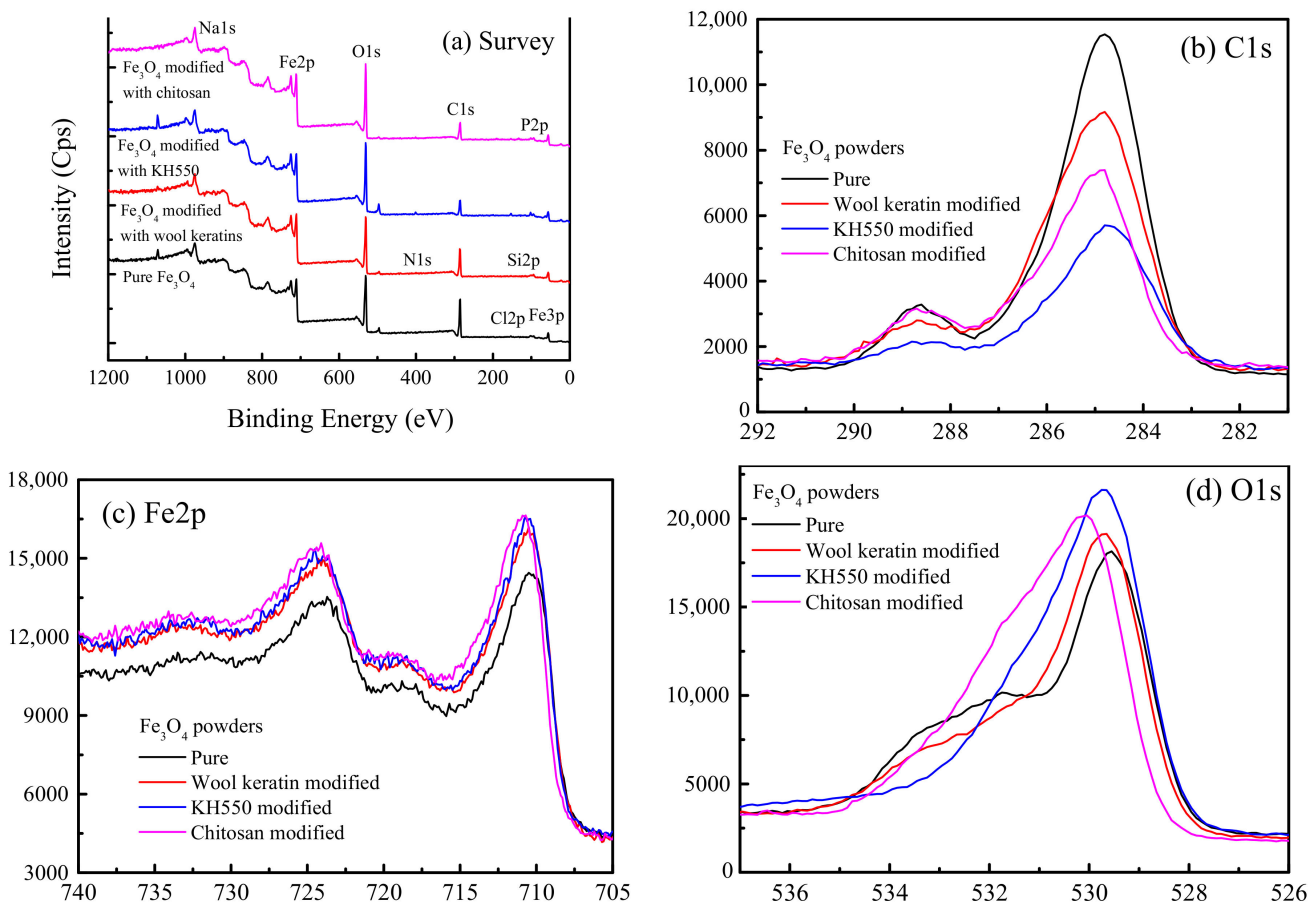
The X-ray photoelectron spectroscopy (XPS) spectra of the pure, wool keratin modified, KH550 modified, and chitosan modified Fe_3_O_4_ powders: (**a**) Survey, (**b**) C1s, (**c**) Fe2p, and (**d**) O1s.

The XPS technique was used to analyze the surface chemical compositions of the pure, wool keratin modified, KH550 modified, and chitosan modified Fe_3_O_4_ powders. The XPS survey spectra and the corresponding analysis data were respectively shown in Figure 4 and Supplementary Materials Table S1. In addition to the elements of C, O, Na, and Fe, a trance amount of Cl element was ascribed to the residual Cl ions of FeCl_2_ or FeCl_3_. The Cl and N elements were identified in the wool keratin modified Fe_3_O_4_ powders besides the elements of C, O, Na, Si, and Fe. In addition to the elements of C, O, Na, Si, and Fe, the Cl element was also detected in the KH550 modified Fe_3_O_4_ powders. After treatment with chitosan, the elements of N and P were detected, which was probably induced by chitosan. At the same time, the elements of Cl and Si disappeared, suggesting chitosan was loaded on the surface of KH550 modified Fe_3_O_4_ powders. The presence of different functional groups like –OH, –COOH, and –NH_2_ from wool keratins resulted in the strong surface complexation with Cu^2+^ ions.

### 3.5. Magnetic Property Analysis

**Figure 5:**
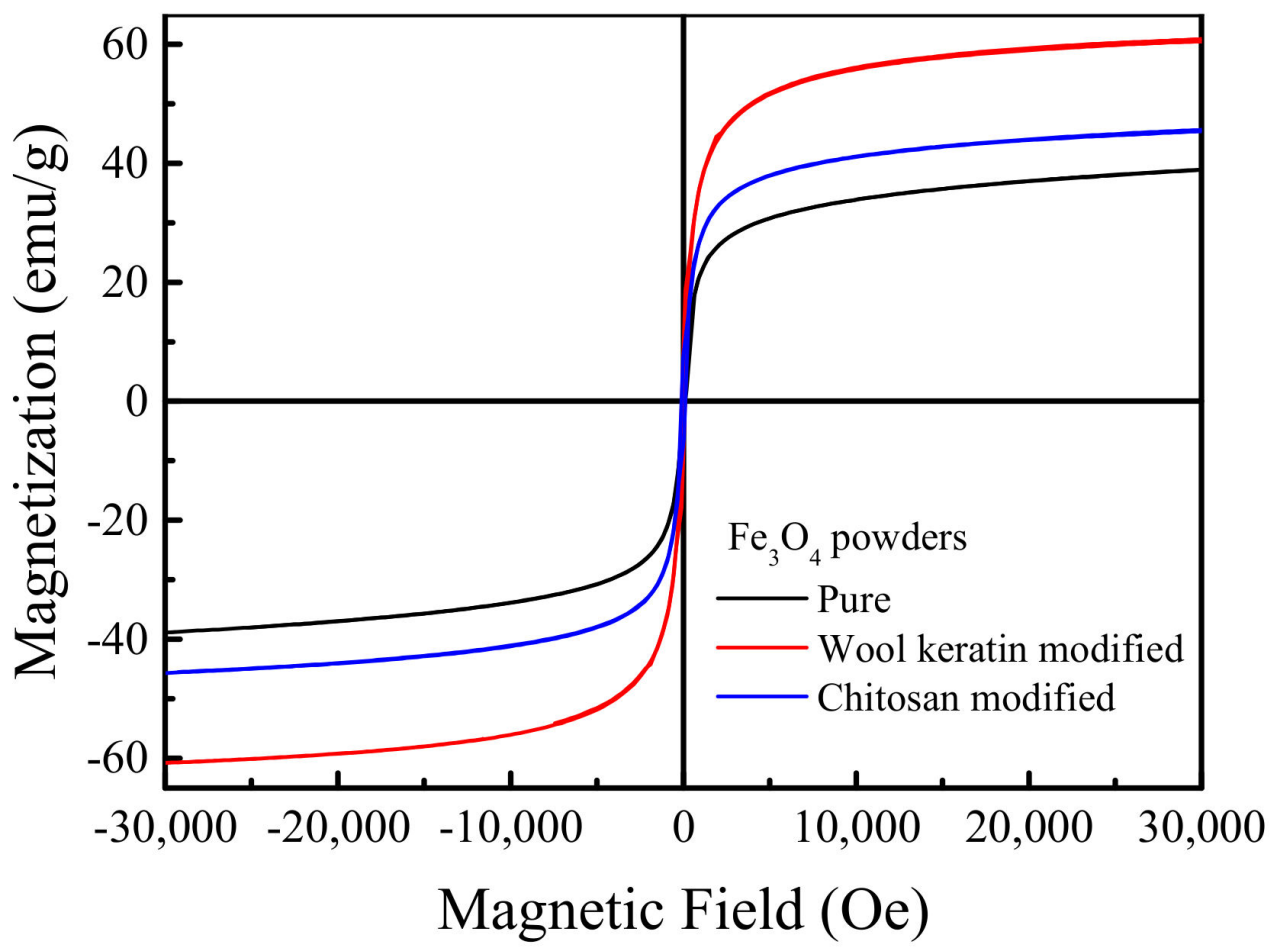
Magnetization curves of the pure, wool keratin modified, and chitosan modified Fe_3_O_4_ powders.

The magnetization curves of the pure, wool keratin modified, and chitosan modified Fe_3_O_4_ powders were depicted in Figure 5. As the applied magnetic field increased, the magnetization quickly increased at first, and then reached a plateau when the magnetic field was beyond 20,000 Oe. The saturation magnetization values were estimated to be 39.2, 60.8, and 45.23 emu/g for the pure, wool keratin modified, and chitosan modified Fe_3_O_4_ powders, respectively [60], larger than 16.3 emu/g as previous reported [61]. Thus, the as-fabricated magnetic powders could be easily separated by using the conventional magnet. All three hysteresis loops passed through the origin and there were no hysteresis phenomena. The remanence and coercivity were zero, indicating they had the superparamagnetic features [62]. The wool keratin modified Fe_3_O_4_ powders possessed the highest saturation magnetization in comparison with the pure and chitosan modified ones. The reason needed to be investigated in future.

### 3.6. Determination of the Contents of Organic Compositions

**Figure 6:**
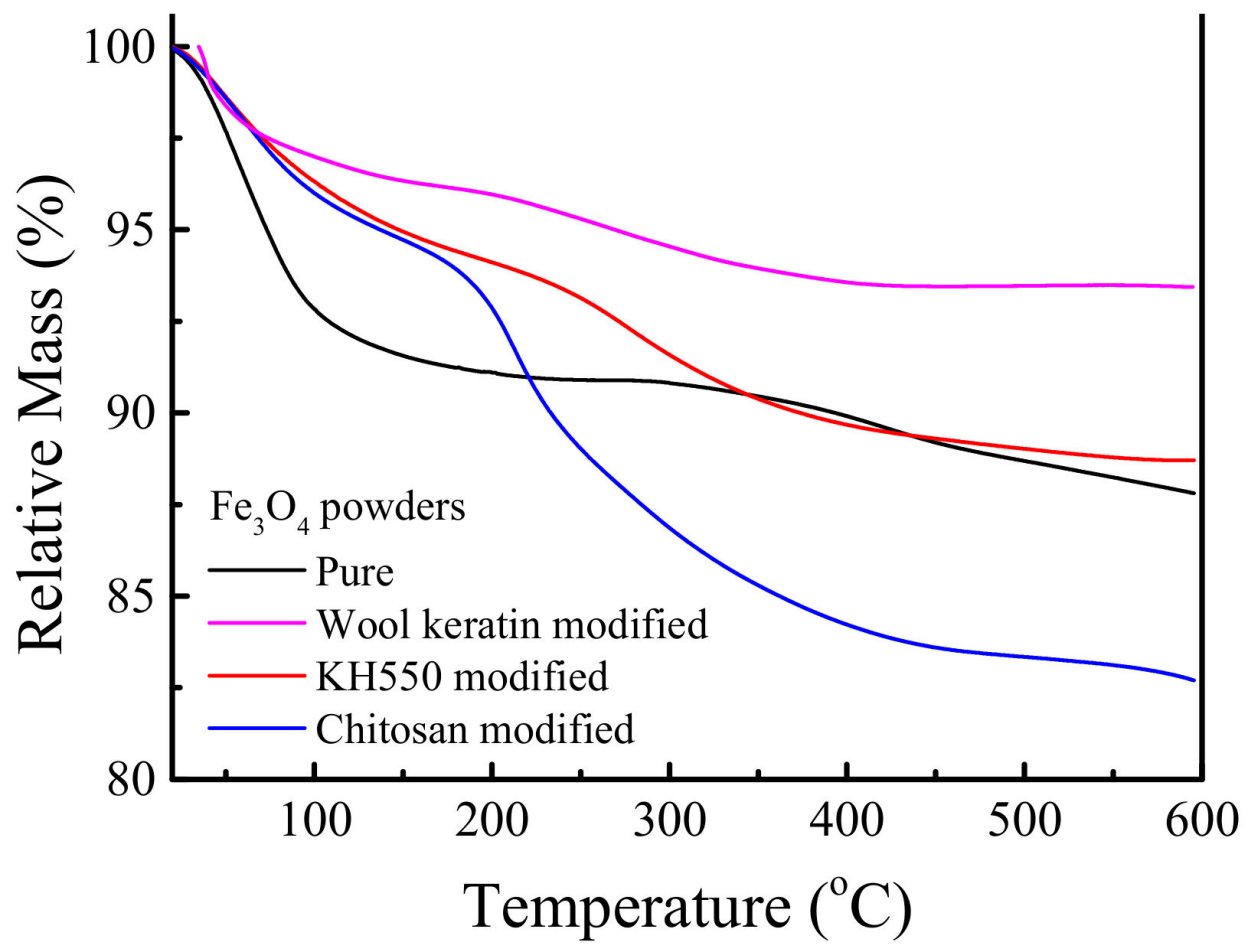
Thermogravimetric (TG) curves of the pure, wool keratin modified, KH550 modified, and chitosan modified Fe_3_O_4_ powders.

The TG analysis was employed to quantify the contents of organic compositions in the as-fabricated Fe_3_O_4_ powders, and the relative mass loss curves versus temperature under nitrogen environment were recorded in Figure 6. It was evident that the pure Fe_3_O_4_ powders contained about 12.2% of organic compositions at 600 °C. There was no significant change in mass after being modified with KH550, about 11.3% of organic compositions was lost. When the KH550 modified Fe_3_O_4_ powders were coated with chitosan, the mass loss of organic compositions was 17.3%. In comparison with the KH550 modified Fe_3_O_4_ powders, about 6% of chitosan was loaded on Fe_3_O_4_ powders, larger than 4.92 wt.% of previous reported [63]. In the case of the wool keratin modified Fe_3_O_4_ powders, 6.6% of organic compositions, namely wool keratins, was lost. Therefore, both wool keratin modified and chitosan modified Fe_3_O_4_ powders had the similar mass percentage of organic compositions.

### 3.7. BET Specific Surface Area and Pore Size Analyses

**Figure 7:**
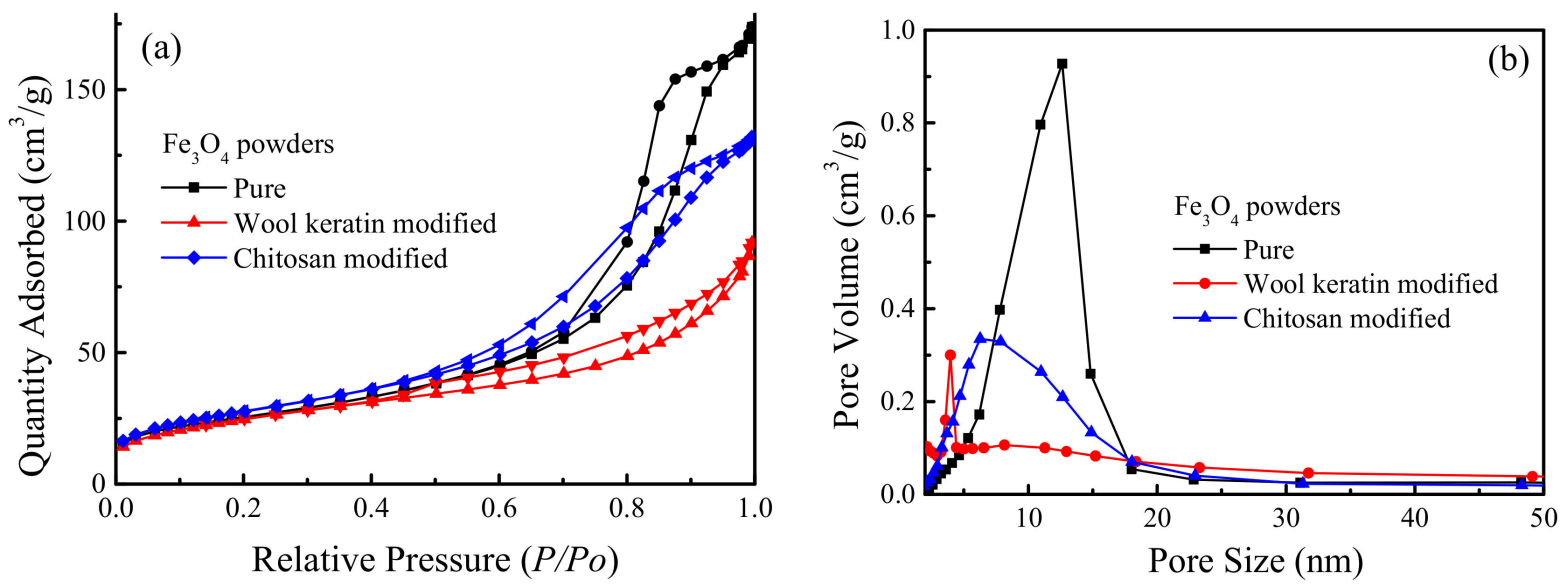
The (**a**) N_2_ adsorption-desorption isotherms and (**b**) pore volume vs. pore size distribution profiles of the pure, wool keratin modified, and chitosan modified Fe_3_O_4_ powders.

The N_2_ adsorption–desorption isotherms and the corresponding pore volume vs. pore size distribution curves of the pure, wool keratin modified, and chitosan modified Fe_3_O_4_ powders were proved in Figure 7. The BET specific surface area of the chitosan modified Fe_3_O_4_ powders increased to 98.5 m^2^/g from 90.1 m^2^/g after being coated with chitosan. However, the BET specific surface area of the wool keratin modified Fe_3_O_4_ powders was slightly reduced to 88.3 m^2^/g when the wool keratin hydrolysate was added during the process of Fe_3_O_4_ synthesis. Based on the International Union of Pure and Applied Chemistry (IUPAC) classification, the sorption isotherms for the three powders belonged to the type IV with the H3 type hysteresis loop in the range of 0.5–1.0 *P*/*P*_0_, which were ascribed to the typical mesoporous materials [64]. The presence of mesopores in the three powders was mainly caused by the accumulation of powders, suggesting the capillary condensation occurring in the mesopores and the existence of fractured pores [65]. The pore volume vs. pore size distribution curves confirmed that the pore size distribution of the pure Fe_3_O_4_ powders was between 5 and 18 nm according to the Barreet–Juyner–Halenda (BJH) method. After modification with chitosan, the range of pore size distribution was not changed but the main pore size was reduced to some degree. For the wool keratin modified Fe_3_O_4_ powders, more smaller pores with sizes of 4 nm or so were formed. Thus, the coating of wool keratins on Fe_3_O_4_ powders made the BET surface area and pore size decrease.

### 3.8. Optimization of the Amount of Wool Keratin Hydrolysate

**Figure 8:**
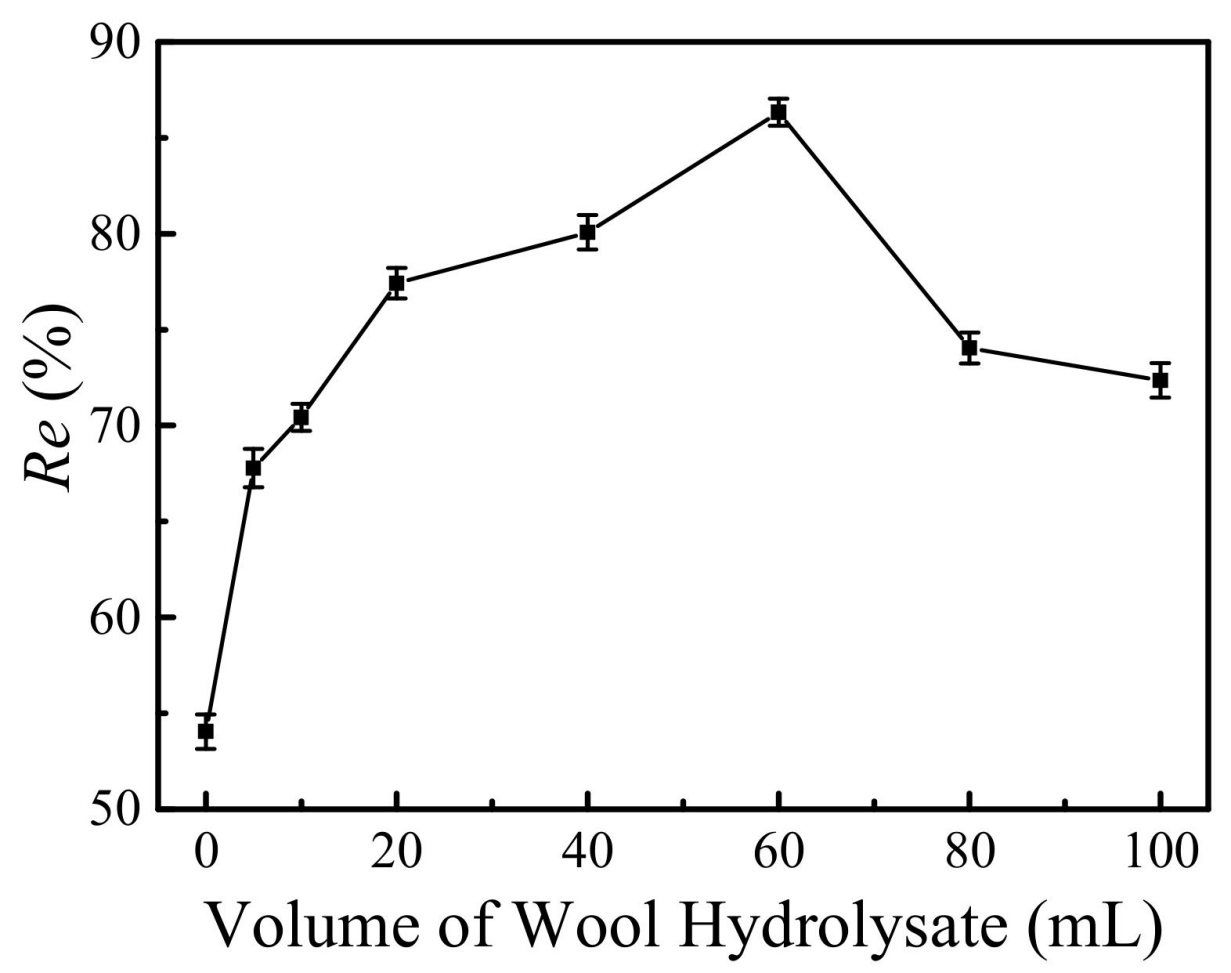
The effects of the volumes of wool keratin hydrolysates on the removal efficiencies of Cu^2+^ ions by the wool keratin modified Fe_3_O_4_ powders.

The effects of the amounts of wool keratin hydrolysates on the removal efficiencies of Cu^2+^ ions onto the wool keratin modified Fe_3_O_4_ powders were disclosed in Figure 8. 0.05 g of powders prepared by using different volumes of wool keratin hydrolysates was dispersed in 50 mL of 10 mg/L CuSO_4_ aqueous solution for 24 h without adjusting the pH value. It was noticed that after being coated by wool keratins, the Cu^2+^ removal capacities for all the wool keratin modified Fe_3_O_4_ powders were enhanced to some degrees. As the volume of wool keratin hydrolysate increased, the removal efficiency of Cu^2+^ ions quickly increased at first. It meant that the coating of wool keratin onto Fe_3_O_4_ powders gradually increased. The highest removal efficiency (about 86%) of Cu^2+^ ions was obtained when 60 mL of wool keratin hydrolysate was added, suggesting an optimum thickness of wool keratin was coated on the surface of Fe_3_O_4_ powders. Afterwards, the removal efficiency of Cu^2+^ ions sharply decreased with increasing the volume of wool keratin hydrolysate. The crystal growth of Fe_3_O_4_ might be restricted or the agglomeration of Fe_3_O_4_ powders probably occurred. Thus, the optimum (60 mL hydrolysate) wool keratin modified Fe_3_O_4_ powders were applied for the following adsorption experiments.

### 3.9. Effect of the Dosage of Powders

**Figure 9:**
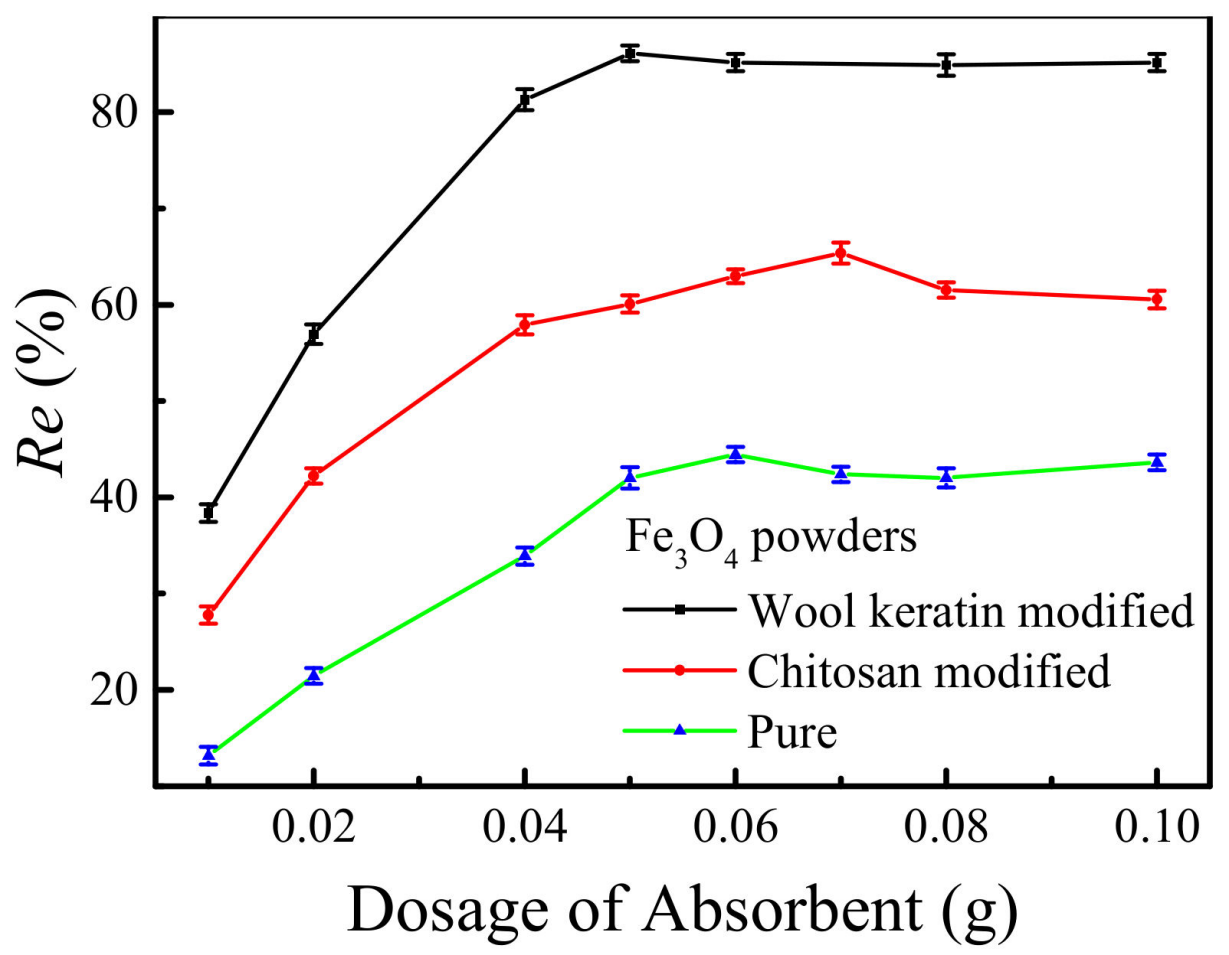
The effects of the dosages of as-fabricated Fe_3_O_4_ powders on the removal efficiencies of Cu^2+^ ions.

The effects of the dosages of as-fabricated powders on the removal efficiencies of Cu^2+^ ions by the pure, wool keratin modified and chitosan modified Fe_3_O_4_ powders were compared in Figure 9. Different dosages (0.01–0.1 g) of magnetic powders were separately dispersed in 50 mL of 10 mg/L CuSO_4_ aqueous solution for 24 h without adjusting the pH value. It was obvious that as the dosage of powders increased, the removal efficiencies of Cu^2+^ ions for the three powders increased significantly. This was because the increased adsorbent numbers provided more active sites for the adsorption of Cu^2+^ ions. Obviously, the wool keratin modified and chitosan modified Fe_3_O_4_ powders behaved better than the pure powders under the same dosage. When the dosages reached 0.05 g and 0.07 g, the highest removal efficiencies of 86% and 65% were obtained onto the wool keratin modified and chitosan modified Fe_3_O_4_ powders, respectively. Moreover, the removal efficiencies of Cu^2+^ ions by the wool keratin modified Fe_3_O_4_ powders were almost larger than those of the chitosan modified Fe_3_O_4_ ones under the same Cu^2+^ ion concentration condition, indicating the strong adsorption capacity of wool keratin modified Fe_3_O_4_ powders to heavy metal ions. Therefore, the optimum dosage (0.05 g) of wool keratin modified Fe_3_O_4_ powders was used for the following studies.

### 3.10. Effect of the Initial Concentration of Cu^2+^ Ions

**Figure 10:**
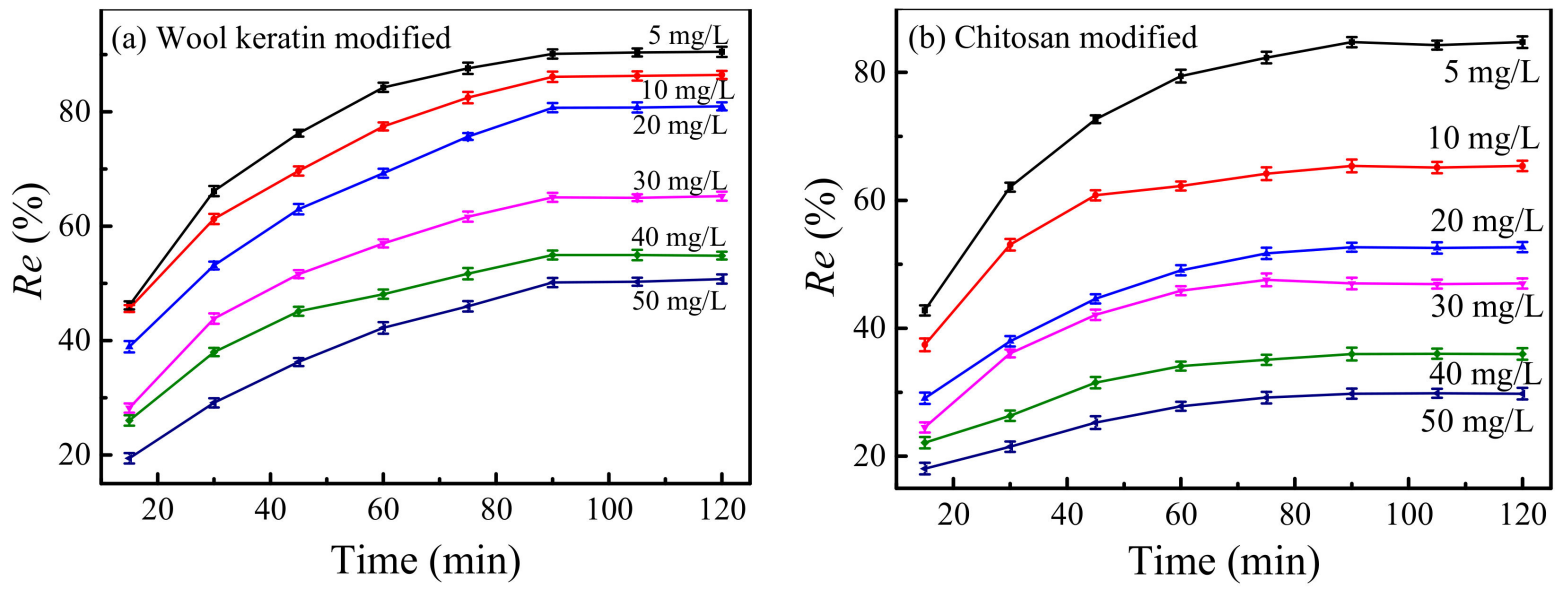
The effects of the initial Cu^2+^ ions on the removal efficiencies of Cu^2+^ ions by the (**a**) wool keratin modified and (**b**) chitosan modified Fe_3_O_4_ powders.

The effects of the concentrations of Cu^2+^ ions on the removal efficiencies of Cu^2+^ ions by the wool keratin modified and chitosan modified Fe_3_O_4_ powders were manifested in Figure 10. 0.05 g of wool keratin modified Fe_3_O_4_ (0.07 g for chitosan modified) powders was separately dispersed in 50 mL of CuSO_4_ aqueous solutions having different concentrations (5–50 mg/L) for different times without adjusting the pH value. It was observed that the removal efficiencies of Cu^2+^ ions increased gradually with the increase of adsorption time, and then reached the adsorption–desorption equilibrium at 90 min for both powders. This was because the accessible active sites on powder surfaces were gradually occupied by more and more Cu^2+^ ions, and thereby there were not enough active sites to bond Cu^2+^ ions. When the concentrations of Cu^2+^ ions were identical, the removal capacity of the wool keratin modified Fe_3_O_4_ powders was superior to that of the chitosan modified ones, confirming the good adsorption performance of wool keratin modified Fe_3_O_4_ powders. Additionally, the removal efficiencies of Cu^2+^ ions for both powders decreased with the increase of initial Cu^2+^ ions. As the initial Cu^2+^ ion concentration increased, more Cu^2+^ ions would compete to combine with the active sites, leading to the insufficient adsorption sites on powder surfaces. Hence, the driving force of Cu^2+^ ions transfer was hindered and the removal efficiencies of Cu^2+^ ions were slow down. The adsorption time was optimized to be 90 min in Cu^2+^ concentrations of 5–50 mg/L.

### 3.11. Effect of the pH Value of Solution

**Figure 11:**
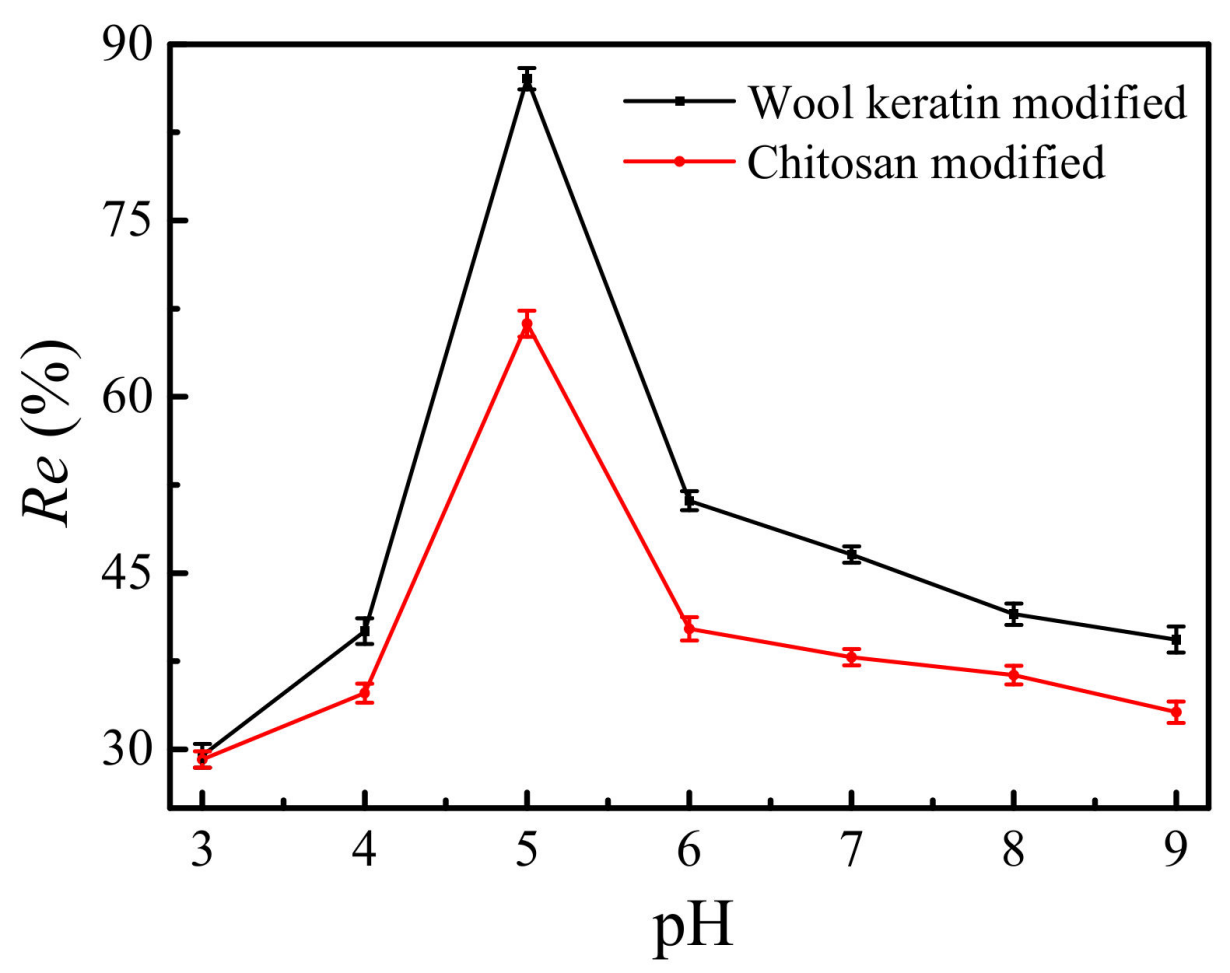
The effects of the pH values of solutions on the removal efficiencies of Cu^2+^ ions by the as-fabricated Fe_3_O_4_ powders.

The effects of the pH values of solutions on the removal efficiencies of Cu^2+^ ions by the wool keratin modified and chitosan modified Fe_3_O_4_ powders were shown in Figure 11. 0.05 g of powders was separately dispersed in 50 mL of 10 mg/L CuSO_4_ aqueous solution for 90 min and the pH values of solutions were adjusted from 3 to 9 using HCl or NaOH. It was apparent that the removal efficiencies for both powders increased with the increase of pH values from 3 to 5 but decreased further with the increase of pH value. This was due to the fact that the pH values of solutions affected the surface charge status of as-fabricated Fe_3_O_4_ powders, the chemical form of Cu^2+^ ions, and the combine process of powders and Cu^2+^ ions [66]. Furthermore, the Visual Minteq program was used to calculate the theoretical Cu species in experimental solutions. The theoretical Cu^2+^ concentration was 1.52 × 10^−4^ mol/L at a pH value of five, larger 1.47 × 10^−4^ at pH = 6 and 7.75 × 10^−5^ mol/L at pH = 7. The Cu ions existed as Cu^2+^ in the solutions at pH 3–5 [67]. However, the Cu(OH)_2_ was formed under basic conditions. The equilibrium concentration of Cu^2+^ ions was measured to be 1.29 mg/L, smaller than 1.30 mg/L of the maximum contaminant level goal for Cu^2+^ in drinking water published by the Environmental Protection Agency’s (EPA) promulgated in 1991. In addition, the adsorption capacity of the wool keratin modified Fe_3_O_4_ powders was higher than that of the chitosan modified ones. As a result, the pH value of solution was set at five.

In addition, the reusability and stability of the as-fabricated powders were evaluated for the potential application. A total of 0.05 g of powders (0.07 g for chitosan modified) was added in 50 mL of 10 mg/L CuSO_4_ aqueous solution at pH 5 for 90 min. The Cu^2+^ ions adsorbed by the powders were desorbed with 0.5 M EDTA–H_2_SO_4_ mixed solution and reused for the next adsorption experiment. The results indicated that about 85–92% of Cu^2+^ ion could be desorbed from the as-modified powders. After five successive Cu^2+^ ion adsorption cycles, the removal efficiency of the wool keratin modified Fe_3_O_4_ powders was maintained above 80%, larger than 52% of the chitosan modified Fe_3_O_4_ powders. Thus, the wool keratin modified Fe_3_O_4_ powders were stable without significant loss of the adsorption capacity towards Cu^2+^ ions and could be applied for the removal of heavy metal ions from aqueous solutions.

### 3.12. Adsorption Isotherms

**Figure 12:**
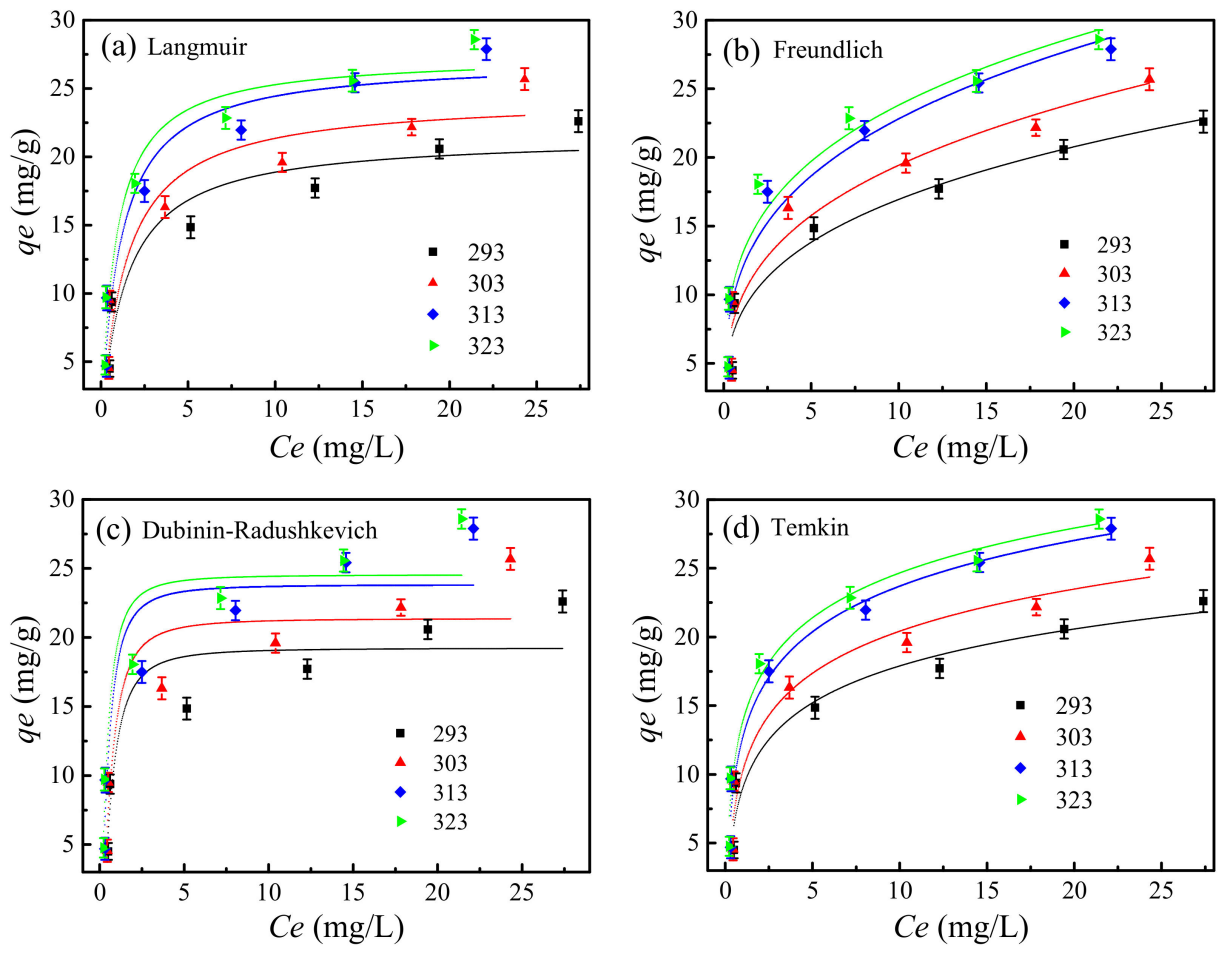
The fitting adsorption isotherm models for (**a**) Langmuir, (**b**) Freundlich, (**c**) Dubinin-Radushkevich, and (**d**) Temkin at different temperatures.

The effects of the initial Cu^2+^ ion concentration on the adsorption capacity of wool keratin modified Fe_3_O_4_ powders were determined by adding 0.05 g powders in a series of flasks containing 50 mL Cu^2+^ ion solution with various concentrations (5, 10, 20, 30, 40, and 50 mg/L) at pH 5. After 90 min adsorption under 293, 303, 313, and 323 K conditions respectively, the supernatants were collected by centrifugation and the residual Cu^2+^ ion concentrations were measured. The adsorption isotherm behaviors were analyzed by using the Langmuir, Freundlich, Dubinin–Radushkevich, and Temkin models, respectively [68]. The fitting adsorption isotherm models under different temperature conditions were plotted in Figure 12. The corresponding fitted data were summarized in Supplementary Materials Tables S2–S6.

#### 3.12.1. Langmuir Isotherm

It was assumed in Langmuir isotherm that the surface of the adsorbents was equal and that every active site could adsorb one molecule of adsorbate [69]. The Langmuir isotherm model could be expressed by the Equation (4) [70] as below:

(4) $$q_{e}=\frac{q_{m}K_{L}C_{e}}{1+K_{L}C_{e}}$$

where *C*_e_ (mg/L) and *q*_e_ (mg/g) were the Cu^2+^ ion concentration in solution and the amount of adsorbed Cu^2+^ ions per gram of the powder both at equilibrium, respectively; *q*_m_ (mg/g) and *K*_L_ (L/mg) were the Langmuir constants which were respectively related to the maximum adsorption capacity and energy of adsorption. It was found in Figure 12a and Supplementary Materials Table S2 that the adsorption capacity of Cu^2+^ ions by the wool keratin modified Fe_3_O_4_ powders increased with the increase of ambient temperature, indicating the adsorption process was endothermic in nature [71].

Langmuir isotherm could be expressed in terms of an equilibrium parameter (*R*_L_) (dimensionless), which was defined by the Equation (5) as below:

(5) $$R_{L}=\frac{1}{1+K_{L}C_{0}}$$

where *C*_0_ (mg/L) was the initial concentration of Cu^2+^ ions. The value of *R*_L_ indicated the type of the isotherm to be either unfavorable (*R*_L_ > 1), linear (*R*_L_ = 1), favorable (0 < *R*_L_ < 1), or irreversible (*R*_L_ = 0). The resultant *R*_L_ values in Supplementary Materials Table S3 were in the range of 0–1, suggesting the favorable adsorption of Cu^2+^ ions onto the wool keratin modified Fe_3_O_4_ powders [6].

#### 3.12.2. Freundlich Isotherm

Based on the adsorption on a heterogeneous surface, the Freundlich isotherm model could be expressed by the Equation (6) [72] as below:

(6) $$q_{e}=K_{F}C_{e}^{1/n}$$

where *K*_F_ ((mg/g)(L/mg)^1/*n*^) was the Freundlich constant and *n* was the heterogeneity factor [66]. It was known that *n* was an empirical parameter that varied with the degree of heterogeneity and was related to the distribution of bonded ions on the sorbent surface. In general, *n* > 1 illustrated that the adsorbate was favorably adsorbed on an adsorbent, and the higher the *n* value the stronger the adsorption intensity [72]. It was noted in Figure 12b and Supplementary Materials Table S4 that the linear fitting *R*^2^ values were close to 1 under different temperature conditions, and all the *n* values were larger than 1, indicating the adsorption of Cu^2+^ ions on the surface of wool keratin modified Fe_3_O_4_ powders was favorable and belonged to the chemical adsorption [73]. The larger the *K*_F_ value, the stronger the adsorption ability of the absorbent. The *K*_F_ value increased with increasing the ambient temperature, meaning that the high temperature was beneficial to the Cu^2+^ ion adsorption onto the wool keratin modified Fe_3_O_4_ powders.

#### 3.12.3. Dubinin–Radushkevich (D–R) Isotherm

The D–R isotherm model was employed to estimate the adsorption performance of microporous adsorbents [70], and could be expressed by the Equation (7) as below:

(7) $$q_{e}=q_{m}\exp(-B{\left[RT\ln(1+\frac{1}{C_{e}})\right]}^{2})$$

where *q*_m_ was the Langmuir constant which was related to the maximum adsorption capacity (mg/g); *B* was the adsorption energy isotherm constant (mol^2^/kJ^2^); *R* was the gas constant (8.314 kJ/kmol·K); *T* was the absolute temperature (K). The free average energy (*E*, kJ/mol) per molecule of the adsorbent could be calculated by the Equation (8) as below:

(8) $$E=\frac{1}{\sqrt{2B}}$$

It was seen in Figure 12c and Supplementary Materials Table S5 that the correlation coefficients at different temperatures were close to 1. All the *E* values were larger than 16 KJ/mol, suggesting that the chemical interactions played an important role in the Cu^2+^ adsorption process onto the wool keratin modified Fe_3_O_4_ powders [74]. However, the results were not agreement with the electrostatic interaction [6] or ion exchange [7] reactions.

#### 3.12.4. Temkin Isotherm

The Temkin isotherm model was used to verify whether the adsorption process was a chemical adsorption with strong electrostatic interactions or not [75]. It could be expressed by the Equation (9) [70] as below:

(9) $$q_{e}=B\ln A+B\ln C_{e}$$

where *A* was the isotherm constant (L/g); *B* = *RT*/*b*, *b* was the adsorption heat constant (kJ/kmol).

It was observed in Figure 12d and Supplementary Materials Table S6 that the Temkin isotherm models under different temperature conditions were fitted the adsorption behavior very well. The correlation coefficients at different temperatures were very close to 1. Thus, the electrostatic interaction was an important factor during the removal of Cu^2+^ ions by the wool keratin modified Fe_3_O_4_ powders [74].

In short, by considering the correlation coefficients of the four different isotherm models under different temperature conditions, the adsorption capacities from the Temkin isotherm model possessed the highest correlation coefficients (*R*^2^ ≥ 0.95), which was not consistent with previous studies [10]. Therefore, the Temkin isotherm model properly exhibited the adsorption behaviors of Cu^2+^ ions, implying that the chemical adsorption with strong electrostatic interactions occurred on the wool keratin modified Fe_3_O_4_ powders [76]. In addition, the maximum adsorption capacity of Cu^2+^ ions from Langmuir equation was 27.4 mg/g at 323 K and the corresponding Langmuir adsorption equilibrium constant was 1.194 L/mg. By comparison with previously-reported data in Supplementary Materials Table S7, it was found that the wool keratin modified Fe_3_O_4_ powders had a higher adsorption capability towards Cu^2+^ ions than the wool keratin nanofibers or wool keratose/silk fibroin blend nanofibers. In comparison with pure Fe_3_O_4_ powders, the adsorption capacity of wool keratin modified Fe_3_O_4_ powders increased by about 1.5 times. However, its adsorption capacity was much lower than the Fe_3_O_4_ chitosan composites and Fe_3_O_4_/poly(L-glutamic acid) microspheres.

### 3.13. Adsorption Kinetics

The adsorption kinetic behaviors of Cu^2+^ ions on the wool keratin modified Fe_3_O_4_ powders were determined by using the pseudo-first-order kinetic, pseudo-second-order kinetic, and intra-particle diffusion models, respectively. A total of 0.05 g of powders was placed in a series of flasks containing 50 mL Cu^2+^ ion solution at a certain initial concentration (5, 10, 20, 30, 40, and 50 mg/L) and a temperature of 293 K. The supernatant in each flask was collected by centrifugation at specific time and the residual Cu^2+^ ion concentration was measured. The fitting adsorption models under different initial Cu^2+^ ion concentration conditions were shown in Supplementary Materials Figure S4. The corresponding fitting parameters were listed in Tables S8–S10. *q*_e,exp_ and *q*_e,cal_ (mg/g) were the adsorption capacities at equilibrium measured experimentally and calculated by the fitting equation, respectively.

#### 3.13.1. Pseudo-First-Order Kinetic Model

The pseudo-first-order kinetic model could be applied for the liquid-phase adsorption kinetic investigation. It was assumed that the diffusion and mass transfer of the adsorbate affected the adsorption rate [74]. The pseudo-first-order kinetic model was expressed by the Equation (10) [73] as below:

(10) $$\frac{dq}{dt}=k_{1}(q_{e}-q_{t})$$

where *t* (min) was the adsorption time; *q*_e_ and *q*_t_ (mg/g) were the adsorption capacities of the powders at equilibrium time and *t* time respectively; *k*_1_ (min^−1^) was the adsorption rate constant of pseudo-first-order kinetic model. It was clear in Supplementary Materials Figure S4a and Table S8 that the linear correlation coefficients for the pseudo-first-order kinetic models were from 0.31 to 0.90 under different initial Cu^2+^ ion concentration conditions, and the calculated *q*_e,cal_ values were close to the experimental *q*_e,exp_ values [77].

#### 3.13.2. Pseudo-Second-Order Kinetic Model

It was believed that the rate-limiting step for the pseudo-second-order kinetic model was a chemical sorption involving valence forces caused by the sharing or exchange of electrons between the adsorbent and the adsorbate [75]. The pseudo-second-order kinetic model could be expressed by the Equation (11) as below:

(11) $$\frac{dq}{dt}=k_{2}{\left(q_{e}-q_{t}\right)}^{2}$$

where *k*_2_ was the adsorption rate constant (g/(mg·min)). The initial adsorption rate (*h*_0_) was defined as: *h*_0_ = *k*_2_*q*_e_^2^ (mg/(g·min)). It was evident in Supplementary Materials Figure S4b and Table S9 that the calculated *q*_e,cal_ values obtained by the pseudo-second-order kinetic model were in well accordance with the experimental *q*_e,exp_ values under different initial Cu^2+^ ion concentration conditions. The correlation coefficients for the pseudo-second-order model were from 0.68 to 0.96. The fitting results indicated the adsorption of Cu^2+^ ions onto the wool keratin modified Fe_3_O_4_ powders was primarily controlled by the chemical adsorption involving valence forces via the electrostatic ion exchange reaction between wool keratin and Cu^2+^ ions in solution [78]. When the concentration of Cu^2+^ ions was 50 mg/L, the initial adsorption rate *h*_0_ increased by 5–6 times. This was mainly induced by the organic functional groups especially –OH^−^ and –COO^−^ anions derived from wool keratins.

#### 3.13.3. Diffusion Model

The Weber–Morris intraparticle diffusion model was used to analyze the control steps in the reaction and to calculate the intraparticle diffusion rate constant of the adsorbent [66]. It was assumed that the effectiveness of the adsorption center and the mass transfer process were the most important factors affecting adsorption kinetics [73]. The Weber–Morris diffusion model could be expressed by the Equation (12) as below:

(12) $$q_{t}=k_{d}t^{1/2}+I$$

where *k*_d_ was the intraparticle diffusion rate constant (mg/g·min^1/2^); *I* was a constant related to the boundary layer thickness (mg/g). As shown in Supplementary Materials Figure S4c and Table S10, because all the *I* values were not equal to zero and the fitted straight lines did not pass through the origin, the intraparticle diffusion was involved in the Cu^2+^ ion adsorption onto the wool keratin modified Fe_3_O_4_ powders. However, the adsorption process was not solely the rate controlling step. Furthermore, the *I* values increased with increasing the initial Cu^2+^ ion concentrations, which promoted the diffusion effect of the boundary layer or the transfer of external mass.

In brief, according to the fitting parameters of above three adsorption kinetic models, the intraparticle diffusion model fitted well with the experimental data, which was not in accordance with the previous studies [10]. The average correlation coefficient of intraparticle diffusion model was higher than those for the other two models. Additionally, the theoretical *q*_e,cal_ values of the pseudo-second-order model were very closer to the experimental *q*_e,exp_ data. Therefore, the intraparticle diffusion and pseudo-second-order kinetic models better described the adsorption of Cu^2+^ ions onto the wool keratin modified Fe_3_O_4_ powders.

### 3.14. Thermodynamic Studies

Given that temperature was essential to the adsorption process, the Gibbs free energy change (Δ*G*°, kJ/mol) for the adsorption of Cu^2+^ ions onto the wool keratin modified Fe_3_O_4_ powders could be expressed by the Equation (13) as below:

(13) $$\Delta G{}^{\circ}=-RT\ln K_{L}$$

where *K*_L_ (L/mg) was the adsorption equilibrium constant, which was obtained from the Langmuir isotherm adsorption equation. At the same time, the relationship among entropy change (Δ*S*°, kJ/mol·K), enthalpy change (Δ*H*°, kJ/mol) and Δ*G*° was expressed by the Equation (14) as below:

(14) ΔG°=ΔH°−TΔS°

Based on the Equations (13) and (14), the Equation (15) was obtained as below:

(15) $$\ln K_{L}=\frac{\Delta S{}^{\circ}}{R}-\frac{\Delta H{}^{\circ}}{RT}$$

Thus, the relationship between *lnK*_L_ and 1/*T* was fitted in Supplementary Materials Figure S5. The fitting parameters including Δ*H*°, Δ*S*° and Δ*G*° were displayed in Table S11. Interestingly, the Δ*G*° values changed from positive to negative when the temperature rose to 323 K, it meant that the adsorption of Cu^2+^ ions onto the wool keratin modified Fe_3_O_4_ powders varied to a spontaneous process from a non-spontaneous process. From Δ*H*° > 0, it indicated that the Cu^2+^ ion adsorption onto the wool keratin modified Fe_3_O_4_ powders was an endothermic reaction. From Δ*S*° > 0, it suggested that the Cu^2+^ ion absorption onto the wool keratin modified Fe_3_O_4_ powders was a process of entropy reduction.

### 3.15. Adsorption Mechanism

**Scheme 2:**
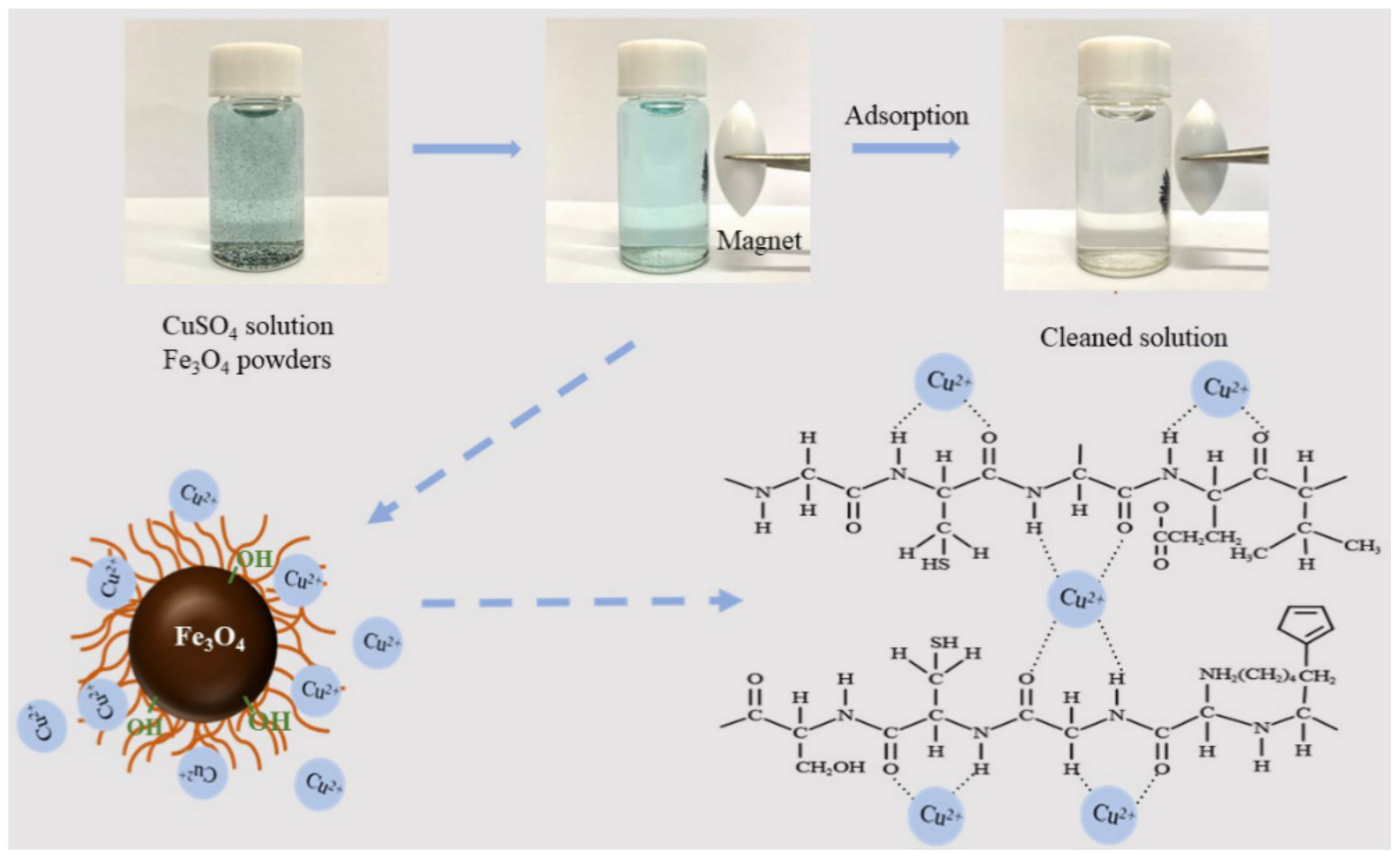
The Cu^2+^ ion adsorption mechanism by the wool keratin modified Fe_3_O_4_ powders.

Based on the above-mentioned structure characterization and Cu^2+^ ion adsorption studies, the proposed adsorption mechanism of the wool keratin modified Fe_3_O_4_ powders was illustrated in Scheme 2. It is known that the water insoluble fibrillar wool keratins are composed of the *α*-spiral polypeptide chains which are chemical bound via the hydrogen bonds and intrachain disulfide cross-links. Under alkaline aqueous solution condition, the disulfide bonds in wools were totally broken because the S element was not detected in the XPS analysis. As demonstrated in Figure S6, wool keratin molecules were chemically grafted onto Fe_3_O_4_ nanoparticles via the C–Fe^3+^/Fe^2+^ and O–Fe^3+^/Fe^2+^ bonds. The functional groups of amide (–CONH) and carboxyl (–COO) from amino acids were formed. These active groups could strongly attract Cu^2+^ ions through the electrostatic interaction and valence forces [15]. On the contrary, the amino groups of chitosan are belonged to a competitive and salt type sorption of metal cations and protons, resulting in the formation of chelate complexes chitosan [(–NH_2_)_2_CuX_2_] with endothermic effect [7]. The mono-functional centers (–NH_2_) of chitosan were distinctly differ from the multi-functional centers of wool keratins with acidic and basic ampholite sorption centers (H_2_N–W–COOH). The zwitter-ionic ^+^H_3_N–W–COO^−^ could be produced in acid aqueous solution. Thus, the anionic carboxyl groups could attract the metal cations and proton, while the cationic ammonium groups could attract the anions [7]. In addition, Fe_3_O_4_ played a certain role in the removal of Cu^2+^ ions. In aqueous solutions, the –FeOH groups presented on Fe_3_O_4_ surfaces, which depended on the pH value of solution, could protonate or deprotonate to FeOH_2_^+^ and FeO^−^. As the pH of solution increased, the amount of FeO^−^ increased to some degree. Fe_3_O_4_ could facilitate the removal of Cu^2+^ by the electrostatic adsorption [79]. As a result, a synergistic interaction between ^+^H_3_N–W–COO^−^ and –FeOH groups promoted the enhanced Cu^2+^ adsorption on the wool keratin modified Fe_3_O_4_ powders.

## 4. Conclusions

The magnetically separable Fe_3_O_4_ powders modified with wool keratins were fabricated during the synthesis process of Fe_3_O_4_ by adding wool keratin hydrolysate. The wool keratin modified magnetic powders could be repeatedly used for the removal of Cu^2+^ ions from aqueous solutions. The experimental results demonstrated that the Cu^2+^ adsorption process was dependent on contact time, initial Cu^2+^ ion concentration, pH value, and temperature of solution. The Cu^2+^ ion adsorption capacity on the wool keratin modified Fe_3_O_4_ powders was higher than that of the chitosan modified ones. The adsorption condition was optimized as follows: 0.05 g of wool keratin modified Fe_3_O_4_ powders were added in 50 mL of 40 mg/L CuSO_4_ solution at pH 5 and temperature 293 K. After 90 min adsorption, the maximum Cu^2+^ adsorption capacity was 27.4 mg/g. Kinetic studies suggested that the adsorption reaction followed the intraparticle diffusion and pseudo-second-order kinetics and the chemical adsorption with strong electrostatic interactions was involved. The adsorption isotherms were well fitted by the Temkin isotherm model. Thermodynamic studies showed that when the temperature rose to 323 K, the Cu^2+^ ion adsorption process on the wool keratin modified Fe_3_O_4_ powders followed a transformation from a non-spontaneous process to a spontaneous process along with the endothermic and entropy reduction effects.

## Data Availability

Data can be available upon request from the authors.

## Supplementary Materials

The following are available online at https://www.mdpi.com/article/10.3390/nano11051068/s1, Figure S1: EDS spectra of the (a) pure, (b) wool keratin modified, (c) KH550 modified, and (d) chitosan modified Fe_3_O_4_ powders, Figure S2: The (a) and (b) TEM, and (c) SAED images, and (d) EDX spectrum along with element analysis data for the pure Fe_3_O_4_ powders, Figure S3: The (a) and (b) TEM, and (c) SAED images, and (d) EDX spectrum along with element analysis data for the chitosan modified Fe_3_O_4_ powders, Figure S4: The fitting adsorption kinetic models for (a) Langmuir, (b) Freundlich, (c) Dubinin-Radushkevich, and (d) Temkin under different initial Cu^2+^ ion concentration conditions, Figure S5: The relationship between *ln*(*K*_L_) and 1/*T* for the Cu^2+^ ion adsorption by the wool keratin modified Fe_3_O_4_ powders, Figure S6: The high-resolution (a) C1s, (b) O1s, (c) N1s, and (d) Fe2p XPS spectra of the wool keratin modified Fe_3_O_4_ powers, Table S1: The surface chemical compositions for the pure, wool keratin modified, KH550 modified, and chitosan modified Fe_3_O_4_ powders, Table S2: The fitting parameters for the Langmuir isotherm model, Table S3: The fitting *R_L_* values for the Langmuir isotherm model, Table S4: The fitting parameters for the Freundlich isotherm model, Table S5: The fitting parameters for the Dubinin–Radushkevich isotherm model, Table S6: The fitting parameters for the Temkin isotherm model, Table S7: The comparison of adsorption capacity of Cu^2+^ ions by different adsorbents, Table S8: The fitting parameters for the pseudo-first-order kinetic model at different Cu^2+^ ion concentrations, Table S9: The fitting parameters for the pseudo-second-order kinetic model at different Cu^2+^ ion concentrations, Table S10: The fitting parameters for the Weber–Morris kinetic model at different Cu^2+^ ion concentrations, Table S11: The fitting thermodynamic parameters at different temperatures.
