# Supplementary material for: Magnetically Recyclable Wool Keratin Modified Magnetite Powders for Efficient Removal of Cu2+ Ions from Aqueous Solutions
Source: Nanomaterials (Basel). 2021 Apr 21;11(5):1068. doi: 10.3390/nano11051068 (PMC8143369; doi:10.3390/nano11051068)
Supplement: Supplementary file 1 [file nanomaterials-11-01068-s001.zip › nanomaterials-1167193-supplementary.pdf]

nanomaterials

Supplementary Materials

Magnetically Recyclable Wool Keratin Modified Magnetite Powders for Efficient Removal of Cu^{2+} Ions from Aqueous Solutions

Xinyue Zhang [1,2,†], Yani Guo [1], Wenjun Li [2,3], Jinyuan Zhang [2,3], Hailiang Wu [3], Ningtao Mao [4] and Hui Zhang [2,3,*,†]

[1] School of Environmental and Chemical Engineering, Xi'an Polytechnic University, Xi'an, Shaanxi 710048, China; 41504040228@stu.xpu.edu.cn (X.Z.); 19940706@xpu.edu.cn (Y.G.)
[2] Research Centre for Functional Textile Materials, School of Textile Science and Engineering, Xi'an Polytechnic University, Xi'an, Shaanxi 710048, China; 200111006@stu.xpu.edu.cn (W.L.); 190121054@stu.xpu.edu.cn (J.Z.); hzhangw532@xpu.edu.cn (H.Z.)
[3] Key Laboratory of Functional Textile Material and Product, Xi'an Polytechnic University, Ministry of Education, Xi'an, Shaanxi 710048, China; whl@xpu.edu.cn
[4] Performance Textiles and Clothing Research Group, School of Design, University of Leeds, Leeds LS2 9JT, UK; n.mao@leeds.ac.uk
* Correspondence: hzhangw532@xpu.edu.cn; Tel.: +86-130029029736
† These authors contributed equally to this work

S1. Fabrication of Chitosan Modified Fe_3O_4 Powders

The pure Fe_3O_4 powders were first prepared using $FeCl_2·4H_2O$ and $FeCl_3·6H_2O$ as the precursors and NaOH as the precipitation agent according to the same method as described in Section 2.2.2. Next, Fe_3O_4 powders were modified with silane coupling agent KH550. 200 mL of mixture solution composed by deionized water and absolute ethanol in volume ratio of 9:1 was prepared. 1% of KH550 in relation to the mass of mixture solution was then added and completely dissolved at ambient temperature under magnetic stirring. A certain amount of Fe_3O_4 powders was subsequently added and sonicated at 28 kHz and 50 W for 30 min based on the ratio of material to liquid 1:30. After 4 h of reaction, the KH550 modified magnetic powders were collected by a magnet and then irradiated for 30 min curing under a 365 nm ultraviolet (UV) lamp. Finally, the UV-cured powders were pre-dried at 110 °C for 30 min and baked at 130 °C for 2 min, and dried in a vacuum oven at 50 °C.

2 mL of acetic acid was first dissolved in 200 mL of deionized water at room temperature. 2 g of chitosan with deacetylation degree >90% and weight average molecular weight 30,000 was then added and treated for 24 h under continuous magnetic stirring to obtain the degraded chitosan. 8% of citric acid and 6% of sodium hypophosphite by mass were successively added in 100 mL of deionized water. After mixing fully, the mixture solution was slowly added into 200 mL of degraded chitosan solution. The as-obtained suspension was sonicated at 28 kHz and 50 W for 30 min. The precipitated black powders were collected by vacuum filtration, and then washed repeatedly with deionized water and absolute ethanol until the pH of solution was neutral, and lastly dried at 80 °C.

S2. The EDS Spectra of the as-Fabricated Fe_3O_4 Powders

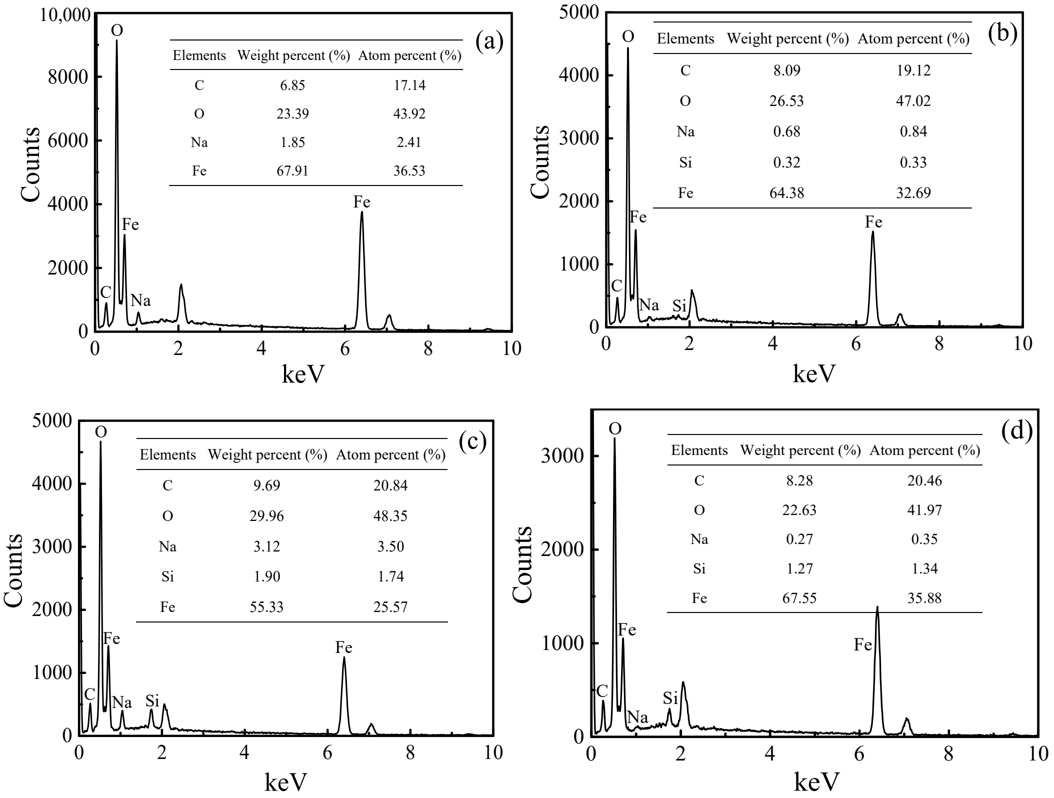

Figure S1. EDS spectra of the (**a**) pure, (**b**) wool keratin modified, (**c**) KH550 modified, and (**d**) chitosan modified Fe_3O_4 powders.

S3. The TEM and SAED Images, and EDX Spectrum for the Pure Fe_3O_4 Powders

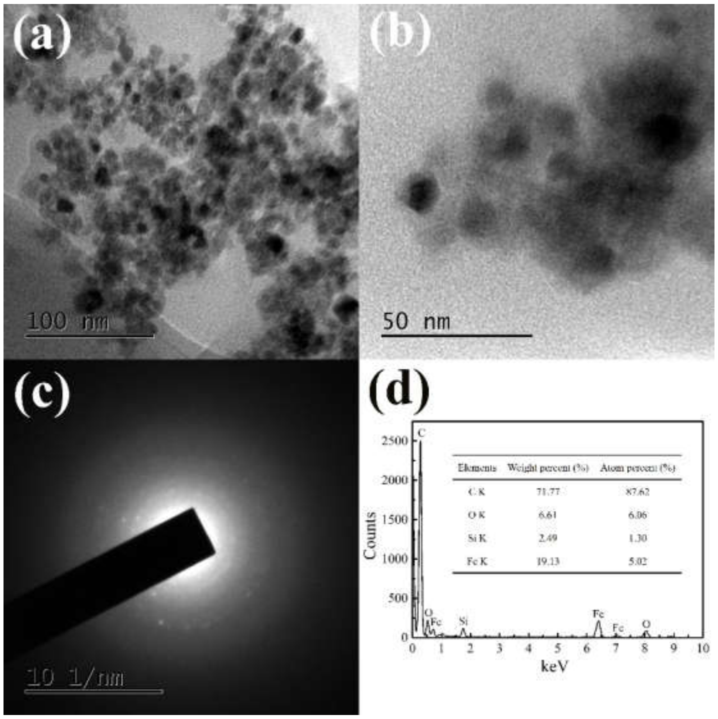

Figure S2. The (**a**) and (**b**) TEM, and (**c**) SAED images, and (**d**) EDX spectrum along with element analysis data for the pure Fe_3O_4 powders.

S4. The TEM and SAED Images, and EDX Spectrum for the Chitosan Modified Fe_3O_4 Powders

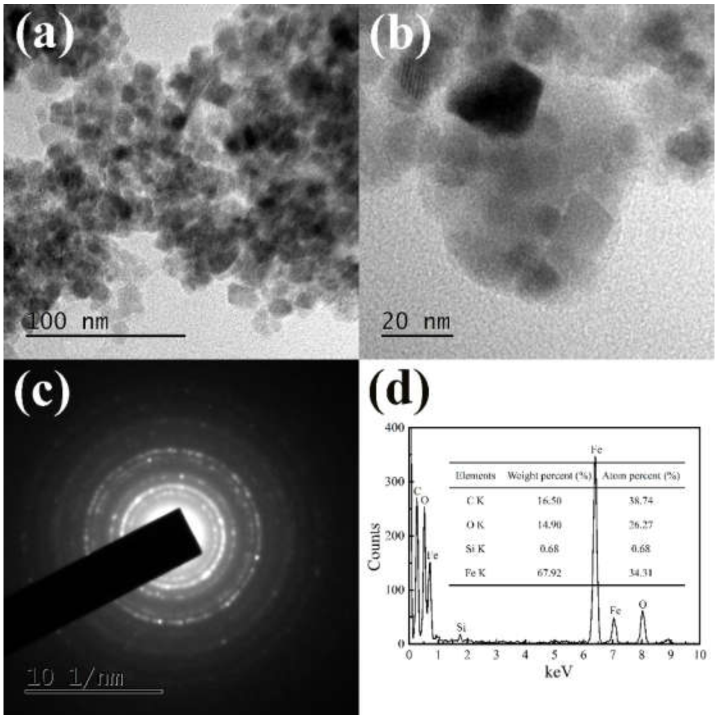

Figure S3. The (**a**) and (**b**) TEM, and (**c**) SAED images, and (**d**) EDX spectrum along with element analysis data for the chitosan modified Fe_3O_4 powders.

S5. The XPS Analysis Results of the as-Fabricated Fe_3O_4 Powders

Table S1. The surface chemical compositions for the pure, wool keratin modified, KH550 modified, and chitosan modified Fe_3O_4 powders.

Fe_3O_4 Powders	Peak	Position BE (eV)	FWHM (eV)	Raw Area (cps.ev)	Atomic Concentration (%)
Pure	C1s	289.51	1.693	26262.6	56.30
	Fe2p	724.55	2.961	42196.3	7.11
	O1s	532.75	1.787	50011.2	35.18
	Na1s	1071.70	1.842	5793.0	1.25
	Cl2p	199.04	2.150	235.0	0.16
Wool keratin modified	C1s	289.35	1.859	21462.2	48.48
	Fe2p	733.51	3.063	63169.1	11.21
	O1s	535.14	1.736	50752.7	37.62
	Na1s	1071.70	1.843	2919.1	0.66
	Cl2p	199.17	2.127	202.4	0.14
	N1s	400.50	1.865	404.4	0.52
	Si2p	101.21	4.784	704.3	1.37
KH550 modified	C1s	289.49	1.889	11742.5	30.47
	Fe2p	732.57	2.855	61621.4	12.56
	O1s	536.04	2.131	57267.9	48.77
	Na1s	1071.82	2.104	12149.9	3.18
	Cl2p	199.11	2.384	453.8	0.37
	Si2p	102.09	2.338	2089.9	4.65
Chitosan modified	C1s	288.99	1.724	16985.3	40.46
	Fe2p	732.50	2.767	60905.2	11.39
	O1s	533.56	1.979	57928.9	45.29
	Na1s	1071.57	1.872	1611.1	0.39
	N1s	400.80	3.496	914.9	1.23
	P2p	133.56	2.174	896.4	1.24

S6. The Fitting Parameters for the Langmuir Isotherm Model

Table S2. The fitting parameters for the Langmuir isotherm model.

Temperature (K)	q_m (mg/g)	K_L (L/mg)	R^2
293	21.503	0.718	0.89
303	24.369	0.712	0.91
313	27.114	0.917	0.93
323	27.400	1.194	0.95

S7. The Equilibrium Parameters (R_L) for the Langmuir Isotherm Model

Table S3. The fitting R_L values for the Langmuir isotherm model**.**

C_0 (mg/L)	R_L			
	**293 K**	**303 K**	**313 K**	**323 K**
5	0.218	0.219	0.179	0.143
10	0.122	0.123	0.098	0.077
20	0.065	0.066	0.052	0.040
30	0.044	0.045	0.035	0.027
40	0.034	0.034	0.027	0.021
50	0.027	0.027	0.021	0.016

S8. The Fitting Parameters for the Freundlich Isotherm Model

Table S4. The fitting parameters for the Freundlich isotherm model**.**

Temperature (K)	n	K_F ((mg/g)(L/mg)$^{1/n}$)	R^2
293	3.403	8.612	0.94
303	3.317	9.711	0.93
313	3.444	11.698	0.94
323	3.679	12.737	0.93

S9. The Fitting Parameters for the Dubinin–Radushkevich Isotherm Model

Table S5. The fitting parameters for the Dubinin–Radushkevich isotherm model.

Temperature (K)	B (10^{-7}, mol^2/kJ^2)	q_m (mg/g)	E (10^3, kJ/mol)	R^2
293	1.659	19.224	1.736	0.82
303	1.410	21.391	1.883	0.82
313	0.920	24.543	2.331	0.84
323	0.710	27.431	2.654	0.87

S10. The Fitting Parameters for the Temkin Isotherm Model

Table S6. The fitting parameters for the Temkin isotherm model**.**

Temperature (K)	A (L/g)	b (kJ/kmol)	R^2
293	10.115	628.646	0.95
303	10.085	568.912	0.95
313	14.083	542.934	0.97
323	18.897	570.638	0.97

S11. Comparison of Adsorption Capacity of Cu^{2+} Ions by Different Adsorbents

Table S7. The comparison of adsorption capacity of Cu^{2+} ions by different adsorbents.

Sample	Cu^{2+} Concentration (g/L)	Adsorbent Dosage (g/L)	q_t (mg/g)	Reference
Wool keratin modified Fe_3O_4	0.05	1.0	27.4	Present work
Wool keratose/silk fibroin blend nanofibrous membranes	0.00349	/	2.88	[5]
Keratin nanofibers	0.1	1.0	20.0	[6]
Keratin nanofiber membranes	0.05	2.14	11.0	[17]
Fe_3O_4 nanoparticles	0.08	2.0	18.6	[68]
Fe_3O_4/poly(L-glutamic acid) microspheres	0.03	0.05	>500	[45]
Magnetic chitosan composites	0.02	0.05	216.8	[S1]

S12. The Fitting Adsorption Kinetic Models

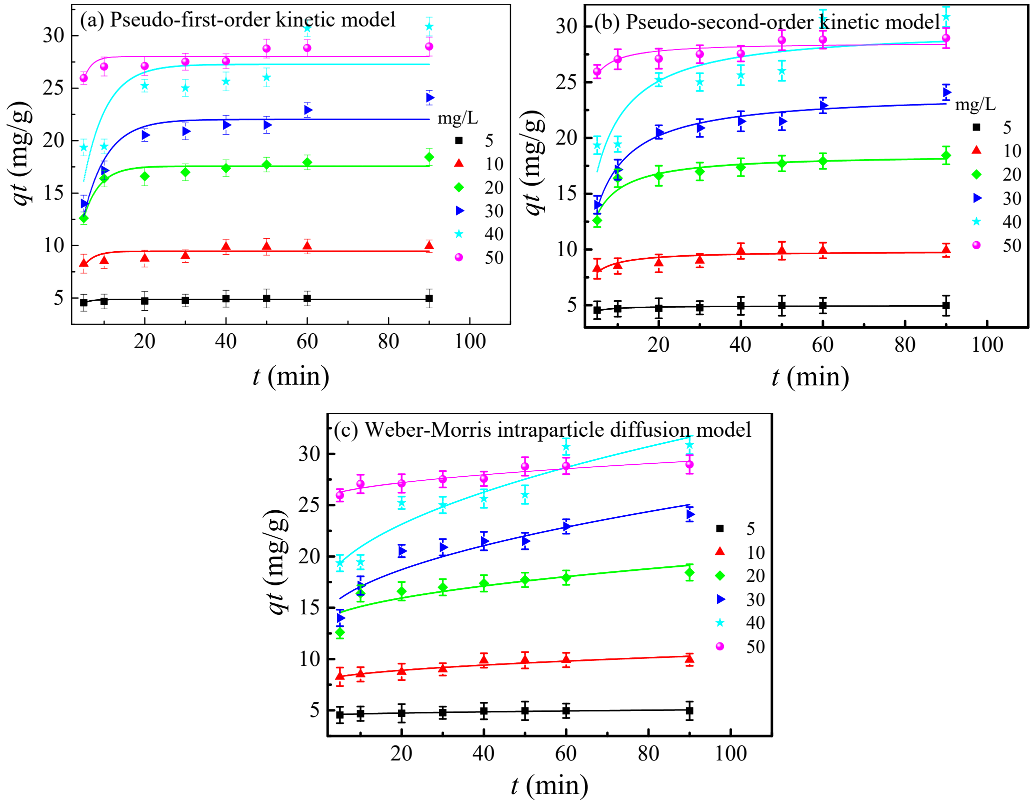

Figure S4. The fitting adsorption kinetic models for (**a**) Pseudo-first-order, (**b**) Pseudo-second-order, and (**c**) Weber-Morris intraparticle diffusion under different initial Cu^{2+} ion concentration conditions.

S13. The Fitting Parameters for the Pseudo-First-Order Kinetic Model

Table S8. The fitting parameters for the pseudo-first-order kinetic model at different Cu^{2+} ion concentrations.

C_0 (mg/L)	$q_{e,exp}$ (mg/g)	$q_{e,cal}$ (mg/g)	k_1 (min^{-1})	R^2
5	4.959	4.859	0.536	0.40
10	9.935	9.471	0.378	0.31
20	18.441	17.561	0.255	0.90
30	24.103	22.024	0.175	0.85
40	30.873	27.266	0.180	0.50
50	28.968	28.018	0.510	0.40

S14. The Fitting Parameters for the Pseudo-Second-Order Kinetic Model

Table S9. The fitting parameters for the pseudo-second-order kinetic model at different Cu^{2+} ion concentrations.

C_0 (mg/L)	$q_{e,exp}$ (mg/g)	$q_{e,cal}$ (mg/g)	k_2 (g/(mg·min))	h_0 (mg/(g·min))	R^2
5	4.959	4.948	0.404	9.881	0.763
10	9.935	9.867	0.083	8.091	0.675
20	18.441	18.547	0.026	8.921	0.917
30	24.103	24.035	0.011	6.345	0.959
40	30.873	29.872	0.009	7.856	0.731
50	28.968	28.577	0.063	51.181	0.697

S15. The Fitting Parameters for the Weber–Morris Kinetic Model

Table S10. The fitting parameters for the Weber–Morris kinetic model at different Cu^{2+} ion concentrations.

C_0 (mg/L)	k_d (mg/g·min$^{1/2}$)	I (mg/g)	R^2
5	0.062	4.458	0.85
10	0.273	7.699	0.85
20	0.636	13.107	0.67
30	1.263	13.070	0.86
40	1.692	15.567	0.88
50	0.415	25.341	0.87

S16. The Fitting Linear Model of *ln*(K_L) versus 1/*T*

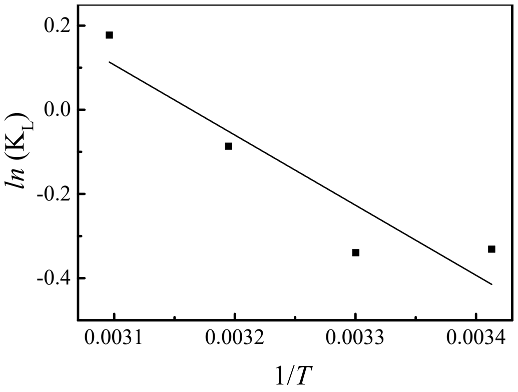

Figure S5. The relationship between *ln*(K_L) and 1/*T* for the Cu^{2+} ion adsorption by the wool keratin modified Fe_3O_4 powders.

S17. The Fitting Thermodynamic Parameters

Table S11. The fitting thermodynamic parameters at different temperatures.

Temperature (K)	ΔG^o (kJ/mol)	ΔH^o (kJ/mol)	ΔS^o (kJ/mol·K)	R^2
293	1.010	13.847	0.043	0.79
303	0.572	/	/	/
313	0.134	/	/	/
323	−0.304	/	/	/

S18. The High-Resolution XPS Spectra of the Wool Keratin Modified Fe_3O_4 Powders

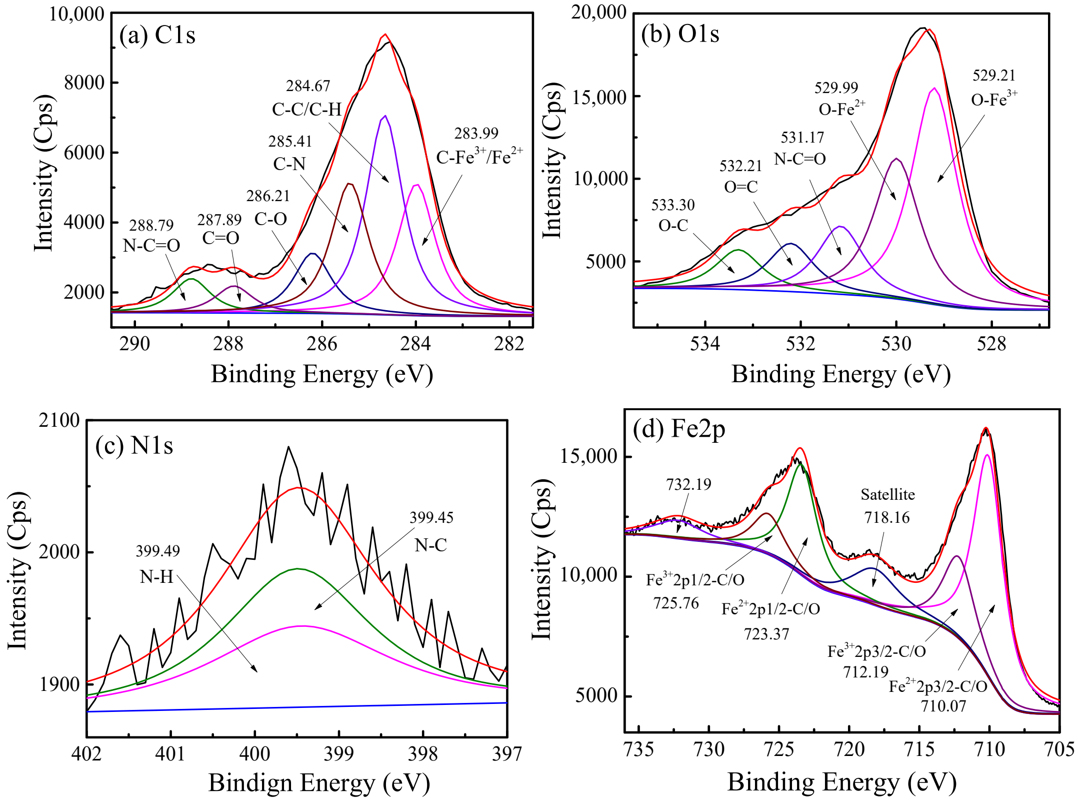

Figure S6. The high-resolution (**a**) C1s, (**b**) O1s, (**c**) N1s, and (**d**) Fe2p XPS spectra of the wool keratin modified Fe_3O_4 powers.

References

[S1]. Peralta, M.E.; Nistico, R.; Franzoso, F.; Magnacca. G. Highly efficient removal of heavy metals from waters by magnetic chitosan-based composite. *Adsorption* **2019**, *25*, 1337–1347.
